# Downregulation of the *ypdA* Gene Encoding an Intermediate of His-Asp Phosphorelay Signaling in *Aspergillus nidulans* Induces the Same Cellular Effects as the Phenylpyrrole Fungicide Fludioxonil

**DOI:** 10.3389/ffunb.2021.675459

**Published:** 2021-09-01

**Authors:** Akira Yoshimi, Daisuke Hagiwara, Miyako Ono, Yasuyuki Fukuma, Yura Midorikawa, Kentaro Furukawa, Tomonori Fujioka, Osamu Mizutani, Natsuko Sato, Ken Miyazawa, Jun-ichi Maruyama, Junichiro Marui, Youhei Yamagata, Tasuku Nakajima, Chihiro Tanaka, Keietsu Abe

**Affiliations:** ^1^New Industry Creation Hatchery Center, Tohoku University, Sendai, Japan; ^2^Laboratory of Environmental Interface Technology of Filamentous Fungi, Kyoto University, Kyoto, Japan; ^3^Medical Mycology Research Center, Chiba University, Chiba, Japan; ^4^Laboratory of Applied Microbiology, Graduate School of Agricultural Science, Tohoku University, Sendai, Japan; ^5^Laboratory of Enzymology, Graduate School of Agricultural Science, Tohoku University, Sendai, Japan; ^6^Department of Biotechnology, Graduate School of Agricultural and Life Sciences, The University of Tokyo, Tokyo, Japan; ^7^Collaborative Research Institute for Innovative Microbiology, The University of Tokyo, Tokyo, Japan; ^8^Terrestrial Microbial Ecology, Graduate School of Agriculture, Kyoto University, Kyoto, Japan

**Keywords:** *Aspergillus nidulans*, His-Asp phosphorelay signaling, YpdA, response regulators, mitogen-activated protein kinases, fludioxonil, growth disorders

## Abstract

Many eukaryotic histidine-to-aspartate (His-Asp) phosphorelay systems consist of three types of signal transducers: a His-kinase (HK), a response regulator (RR), and a histidine-containing phosphotransfer intermediate (HPt). In general, the HPt acts as an intermediate between the HK and the RR and is indispensable for inducing appropriate responses to environmental stresses. In a previous study, we attempted but were unable to obtain deletion mutants of the *ypdA* gene in order to characterize its function in the filamentous fungus *Aspergillus nidulans*. In the present study, we constructed the C*ypdA* strain in which *ypdA* expression is conditionally regulated by the *A. nidulans alcA* promoter. We constructed C*ypdA* strains with RR gene disruptions (C*ypdA-sskA*Δ, C*ypdA-srrA*Δ, and C*ypdA-sskA*Δ*srrA*Δ). Suppression of YpdA induced by *ypdA* downregulation activated the downstream HogA mitogen-activated protein kinase cascade. YpdA suppression caused severe growth defects and abnormal hyphae, with features such as enhanced septation, a decrease in number of nuclei, nuclear fragmentation, and hypertrophy of vacuoles, both regulated in an SskA–dependent manner. Fludioxonil treatment caused the same cellular responses as *ypdA* suppression. The growth-inhibitory effects of fludioxonil and the lethality caused by *ypdA* downregulation may be caused by the same or similar mechanisms and to be dependent on both the SskA and SrrA pathways.

## Introduction

Histidine-to-aspartate (His-Asp) phosphorelay (or two-component) systems are common in bacteria, slime molds, plants, and fungi and are involved in a wide variety of cellular responses to environmental stimuli (Hoch and Silhavy, 1995). These systems commonly consist of (i) a His-kinase (HK) that serves as a sensor for certain stimuli, (ii) a response regulator (RR) containing a phospho-accepting receiver domain, and (iii) a histidine-containing phosphotransfer intermediate (HPt). Eukaryotic HKs are generally hybrid-type kinases that contain a kinase and a receiver domain. A phosphate group is transmitted as follows: hybrid HK (His) → hybrid HK (Asp) → HPt (His) → RR (Asp). This type of sequential phosphorelay is generally referred to as a multistep phosphorelay (Appleby et al., 1996). Numerous instances of such His-Asp phosphorelay systems have been identified and characterized in many fungi (Maeda et al., 1994; Alex et al., 1996; Yamada-Okabe et al., 1999; Buck et al., 2001). In a previous study, we focused on the His-Asp phosphorelay system of a model filamentous fungus, *Aspergillus nidulans*, which is a representative of related species of interest, such as *A. fumigatus* (a serious pathogen) and *A. oryzae* (a fungus used for industrial fermentation). Based on genome sequence data, *A. nidulans* has 15 HKs, four RRs, and only one HPt (Hagiwara et al., 2007a). The physiological functions of four HKs (TcsA, TcsB, FphA, and NikA) and four RRs (SskA, SrrA, SrrB, and SrrC) have been investigated so far (Appleyard et al., 2000; Furukawa et al., 2002; Blumenstein et al., 2005; Hagiwara et al., 2007a,b). The two-component signaling system in *A. nidulans* is summarized in Supplementary Figure 1.

In *Saccharomyces cerevisiae*, the His-Asp phosphorelay system has been intensively studied by many researchers. *Saccharomyces cerevisiae* has a single transmembrane HK, Sln1p; one HPt, Ypd1p; and two RRs, Ssk1p and Skn7p. A typical multistep phosphorelay pathway consisting of Sln1p (hybrid HK)–Ypd1p (HPt)–Ssk1p (RR) is crucial to the osmotic stress response; this pathway modulates the downstream high osmolarity glycerol (HOG) mitogen-activated protein kinase (MAPK) cascade consisting of Ssk2p/Ssk22p (MAPKKK), Pbs2p (MAPKK), and Hog1p (MAPK) (Maeda et al., 1994; Posas et al., 1996; Posas and Saito, 1998). Thus, when cells are exposed to high osmotic stress, the HOG MAPK pathway is activated *via* the His-Asp phosphorelay system. Activation of the HOG pathway results in the induction of genes required for osmotic adaptation, including glycerol biosynthesis genes such as glycerol-3-phosphate dehydrogenase (*GPD1*) and glycerol-3-phosphatase (*GPP2*) (Akhtar et al., 1997; Gustin et al., 1998; Hohmann, 2002). In *S. cerevisiae*, the RR Skn7p is also regulated *via* Sln1p-Ypd1p (Li et al., 1998). Skn7p is a transcription factor involved in multiple physiological processes, such as maintenance of cell wall integrity and oxidative stress response (Brown et al., 1993, 1994).

Based on these findings for *S. cerevisiae*, the signaling network of the His-Asp phosphorelay system and the HOG MAPK cascade were investigated in the filamentous fungus *A. nidulans*. When *A. nidulans* cells are exposed to high osmotic stress, HogA (Hog1 ortholog), also called SakA, is phosphorylated in a manner dependent on PbsB, SskB, and SskA (orthologs of Pbs2p, Ssk2p/Ssk22p, and Ssk1p, respectively), but not on TcsB (an ortholog of Sln1p) (Furukawa et al., 2005). Deletion mutants of HogA and SskA are similarly sensitive to osmotic stress (Furukawa et al., 2005), and their conidia are sensitive to oxidative and heat stress (Kawasaki et al., 2002; Hagiwara et al., 2007a). The HogA MAPK cascade is involved in light sensing *via* the interaction between the phytochrome FphA and the HPt protein YpdA in *A. nidulans* (Yu et al., 2016). In response to white or red light, SakA (HogA) translocates from the cytoplasm to the nucleus in an FphA-dependent manner (Yu et al., 2016). FphA and TcsB are also involved in temperature sensing (Yu et al., 2019). FphA could act as a thermosensor and is involved in SakA translocation, although the temperature response of SakA is not completely FphA dependent (Yu et al., 2019). Thus, the His-Asp phosphorelay system appears to regulate the HogA MAPK cascade and to play a specific role in *A. nidulans* response to environmental changes (Supplementary Figure 1). Through this signaling network, genes encoding stress-related factors, such as glycerol-3-phosphate dehydrogenase (GfdB) (Han and Prade, 2002), trehalose-6-phosphate synthase (TpsA), and conidia-specific catalase (CatA) (Navarro and Aguirre, 1998), are regulated in response to osmotic stress (Hagiwara et al., 2007a, 2009). The light- and temperature-regulated genes, *conJ, ccgA*, and *ccgB*, are also regulated in a HOG pathway–dependent manner (Yu et al., 2016, 2019). Previously, we performed transcriptome analyses using DNA microarrays, which revealed that the His-Asp phosphorelay system and the HogA MAPK cascade are responsible for the transcriptional responses to both fungicide and osmotic stress in *A. nidulans* (Hagiwara et al., 2009).

Phenylpyrrole- or dicarboximide-type fungicides such as fludioxonil or iprodione, respectively, are agronomically useful. These fungicides appear to act *via* the signaling network of the His-Asp phosphorelay system and the HOG MAPK cascade (Ochiai et al., 2001; Yoshimi et al., 2004, 2005; Motoyama et al., 2005; Viaud et al., 2006; Hagiwara et al., 2007b). Fludioxonil converts Drk1 (an HK from *Blastomyces dermatitidis*, expressed in *S. cerevisiae*) into a phosphatase, resulting in Ypd1 dephosphorylation and constitutive activation of the HOG pathway (Lawry et al., 2017). Brandhorst et al. (2019) have suggested the molecular mechanism of this conversion. Fludioxonil could modify the action of triosephosphate isomerase, inducing elevation of methylglyoxal in the Drk1-expressing yeast. Methylglyoxal stress could act on Drk1 and convert it to a phosphatase, inducing constitutive activation of the HOG pathway and cell death (Brandhorst et al., 2019).

To better understand the molecular mechanisms of the His-Asp phosphorelay system, we attempted to characterize the *A. nidulans* HPt, YpdA. Despite many attempts to obtain a *ypdA* disruptant, we could not isolate it (Furukawa et al., 2005). Because deletion of Ypd1p is lethal in *S. cerevisiae*, deletion of *A. nidulans* YpdA was also presumed to be lethal (Posas et al., 1996). Vargas-Perez et al. (2007) also tried to obtain an *A. nidulans ypdA* gene deletion strain and obtained *ypdA*^−^*/ypdA*^+^ heterokaryons, but could not segregate the desired *ypdA*^−^ haploid strain, demonstrating that the *ypdA*^−^ allele could only be maintained in heterokaryons. Vargas-Perez et al. (2007) concluded that *ypdA* deletion is probably lethal in *A. nidulans*. In *Neurospora crassa*, the *hpt-1* gene encoding a single ortholog of Ypd1p could be disrupted in the *os-2* (*hog1* ortholog gene) mutant but not in the wild-type strain (Banno et al., 2007), indicating that the HPt gene is essential in wild-type *N. crassa*, but not in the *os-2* mutant background. Collectively, these data indicate that HPt may be an essential component and may play a crucial role in His-Asp phosphorelay signaling systems in filamentous fungi. Although many studies have suggested that *ypdA* and its homologs are essential in many species of fungi, as described above, the involvement of elements downstream of YpdA remains unclear.

In filamentous fungi such as *A. nidulans* (Hagiwara et al., 2007b) and *Cochliobolus heterostrophus* (Yoshimi et al., 2004), resistance to the fungicide fludioxonil is known to depend on two-component signaling. Fludioxonil strongly inhibits growth of wild-type strains of these two fungi, and mutants with the two RR genes disrupted (*sskA*Δ*srrA*Δ) are fully resistant to fludioxonil (Hagiwara et al., 2007b; Izumitsu et al., 2007). Because fludioxonil treatment increases the levels of phosphorylated Hog1-type MAP kinases (Kojima et al., 2004), the toxicity of fludioxonil and the lethality of *ypdA* disruption, which is thought to cause hyper-activation of HogA, may share some components of their mechanisms (Furukawa et al., 2005; Vargas-Perez et al., 2007). Although the essentiality of the *ypdA* gene and the toxicity of fludioxonil are known, as described above, the detailed intracellular events caused by *ypdA* downregulation or fludioxonil treatment remain unclear.

In the present study, we constructed a strain of *A. nidulans* in which *ypdA* was conditionally regulatable, with or without deletion of *sskA* and/or *srrA*, which encode RRs in the two-component signaling system. We downregulated expression of *ypdA* in each strain and demonstrated that *ypdA* downregulation causes severe growth defects and abnormal hyphae, with features such as enhanced septation, a decrease in number of nuclei, and hypertrophy of vacuoles. The growth defects caused by *ypdA* downregulation were restored by *sskA*Δ*srrA*Δ double disruption. Fludioxonil treatment of wild-type cells produced phenotypes similar to those caused by *ypdA* downregulation. The intracellular phenomena caused by *ypdA* downregulation and fludioxonil treatment are discussed from the viewpoint of the events downstream of YpdA signaling.

## Results

### Construction of the *alcA* Promoter-Driven *ypdA* Strain (C*ypdA*) and Its Response Regulator Gene Disruptants (C*ypdA*-*sskA*Δ, C*ypdA*-*srrA*Δ, and C*ypdA*-*sskA*Δ*srrA*Δ Strains)

To assess the essentiality of the *ypdA* gene and investigate the physiological role of YpdA in *A. nidulans*, we generated a strain, in which the transcription of *ypdA* was under the control of the *A. nidulans alcA* promoter. The native *ypdA* promoter (−1,000 bp from the start codon) was replaced by the *alcA* promoter and the *pyrG* selectable marker in the ABPU1 (Δ*ligD*) strain (Supplementary Figure 2, upper panel). The genomic structure of the resulting conditional-*ypdA* (C*ypdA*) strain was confirmed by PCR (Supplementary Figure 2, lower panel). From the C*ypdA* strain, we constructed *sskA* single disruptants (C*ypdA*-*sskA*Δ), *srrA* single disruptants (C*ypdA*-*srrA*Δ), and *sskA*-*srrA* double disruptants (C*ypdA*-*sskA*Δ*srrA*Δ). Two other RR genes of *A. nidulans, srrB* and *srrC*, were excluded from the present study because deletion of either of these genes had no effect on phenotype (Hagiwara et al., 2007a). Disruption of the *sskA* and *srrA* genes in the C*ypdA*-*sskA*Δ, C*ypdA*-*srrA*Δ, and C*ypdA*-*sskA*Δ*srrA*Δ strains was confirmed by PCR (Supplementary Figure 3).

### Regulation of the Expression Level of the *ypdA* Gene

To confirm the regulation of *ypdA* expression in the C*ypdA* strain and its derivatives C*ypdA*-*sskA*Δ, C*ypdA*-*srrA*Δ, and C*ypdA*-*sskA*Δ*srrA*Δ, we performed transcriptional analysis. The *alcA* promoter activates gene expression in the presence of carbon sources such as ethanol, glycerol, and threonine, and represses it in the presence of glucose (Gwynne et al., 1987). In addition, the presence of yeast extracts often affects the expression of genes controlled by the *alcA* promoter (Nikolaev et al., 2002). First, the composition of CD medium was optimized to produce CDTFY medium (50 mM threonine, 0.2% fructose, 0.2% yeast extract) to decrease the transcription levels of *ypdA* in the C*ypdA* strain to less than three times that in the wild-type strain ABPU1 (Supplementary Figure 4A). Next, using this medium, we evaluated the relative expression levels of *ypdA* in C*ypdA* and its derivative strains, and found that they did not differ significantly among strains under these growth conditions (Supplementary Figure 4B).

**Figure 1:**
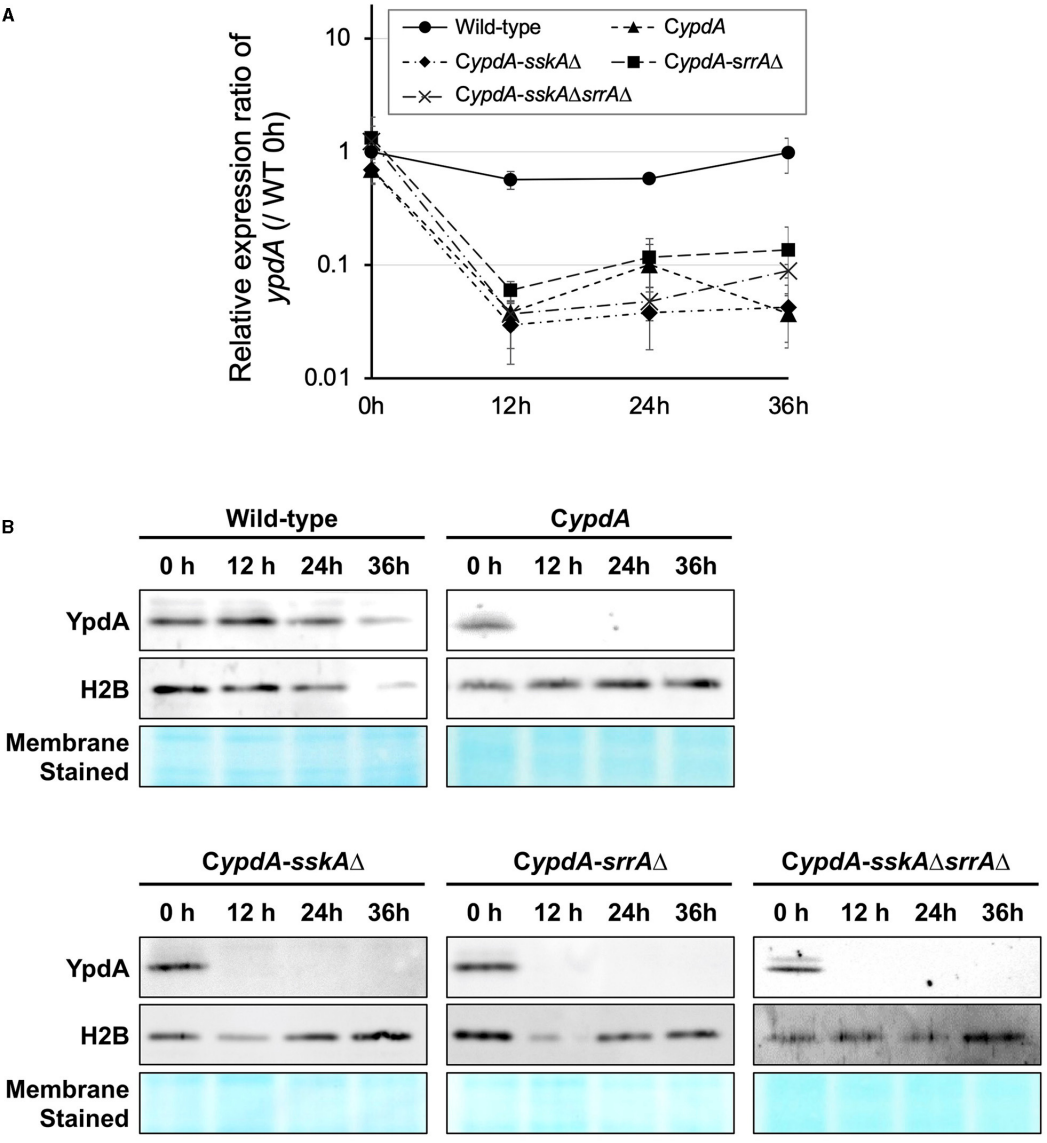
Downregulation of the *ypdA* gene **(A)** and YpdA protein levels **(B)** in *Aspergillus nidulans* wild-type, C*ypdA*, and C*ypdA*-derivative strains. **(A)** Time course of *ypdA* expression after transfer from *ypdA*-inducing medium (CDTFY; 0 h) to *ypdA*-repressing medium (CDY). Conidia (final concentration, 5 × 10^5^ conidia/ml) were inoculated into *ypdA*-inducing medium and cultured for 20 h before transfer to *ypdA*-repressing medium, at 0 h. Samples were prepared from mycelia at 12, 24, and 36 h, and *ypdA* expression was quantified by RT-PCR. The expression level of the *ypdA* gene in each sample was normalized to that of the histone H2B gene (internal control) in the same sample. Each value represents the *ypdA* expression ratio relative to expression in the wild-type strain cultured in CDTFY medium for 20 h (WT 0 h). Error bars represent standard deviations from three independent replicates. **(B)** Western blot analysis of YpdA protein with histone H2B protein as an internal standard and stained membrane for confirmation of protein transfer. Samples were prepared from mycelia at 12, 24, and 36 h after transfer from *ypdA*-inducing medium (CDTFY; 0 h) to *ypdA*-repressing medium (CDY). Each lane was loaded with 15 μg protein.

To repress the transcription of *ypdA*, CDY medium (2% glucose, 0.1% yeast extract) was used for cultures of C*ypdA* and its derivatives. Transcription levels of *ypdA* in the wild-type strain remained stable for 36 h after transfer from CDTFY (0 h) to CDY (Figure 1A). In contrast, after 12 h on CDY medium, transcription levels of *ypdA* in the C*ypdA* and its derivative strains were ≤10% of those in the wild-type strain (Figure 1A). Two-way ANOVA revealed a significant interaction between strain and time; thus, multiple-comparison tests of the *ypdA* expression level were conducted. At each time point from 12 h after transfer to *ypdA*-repressing medium (CDY), the expression levels of *ypdA* in all the mutant strains were significantly lower than those in the wild-type strain (Supplementary Table 1). To confirm that *ypdA* expression levels affected YpdA protein levels, we performed western blot analysis using an anti-YpdA antibody (Figure 1B). After transfer from *ypdA*-inducing to *ypdA*-repressing medium, YpdA protein was hardly detectable in C*ypdA* and its derivative strains.

Thus, in *ypdA*-repressing medium, both *ypdA* transcription and YpdA protein levels were stable in the wild type but were downregulated in C*ypdA* and its derivative strains.

### Downregulation of *ypdA* Induces HogA Pathway–Regulated Genes

**Figure 2:**
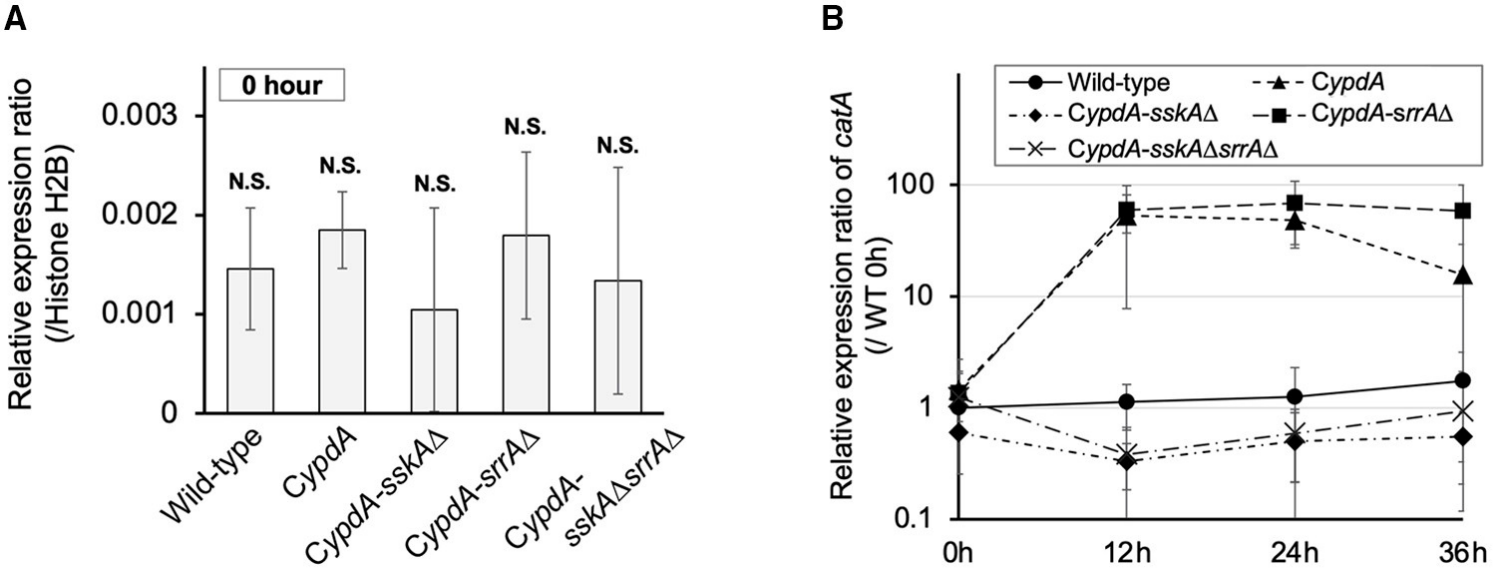
Expression levels of *catA* in *Aspergillus nidulans* wild-type, C*ypdA*, and C*ypdA*-derivative strains. **(A)** Quantitative real-time PCR analysis. Conidia (final concentration, 5 × 10^5^ conidia/ml) were inoculated into *ypdA*-inducing CDTFY medium and cultured for 20 h before transfer to *ypdA*-repressing CDY medium, at 0 h. Transcript levels of *catA* were quantified by RT-PCR. Each value represents the expression ratio relative to the gene encoding H2B in each strain. Error bars represent standard deviations from three independent replicates. N.S., not significant. **(B)** Time course of *catA* expression after transfer from *ypdA*-inducing medium (CDTFY; 0 h) to *ypdA*-repressing medium (CDY). Samples were prepared from mycelia at 12, 24, and 36 h, and expression of *catA* was quantified by RT-PCR. The expression level of the *catA* gene in each sample was normalized to that of the histone H2B gene (internal control) in the same sample. Each value represents the *catA* expression ratio relative to expression in the wild-type strain cultured in CDTFY medium for 20 h (WT 0 h). Error bars represent standard deviations from three independent replicates.

In His-Asp phosphorelay systems, HPt factors mediate phosphotransfer between HKs and RRs, so we investigated whether a decreased level of YpdA affects downstream signaling pathways. Because the HogA MAPK cascade is regulated downstream of SskA, one of the RRs of interest, we investigated the expression of the HOG pathway–regulated gene *catA* (Hagiwara et al., 2007a). On *ypdA*-inducing (CDTFY) medium, no significant differences were detected in the relative expression ratio of *catA* among strains (Figure 2A). After transfer to *ypdA*-repressing medium, transcription levels of *catA* tended to be upregulated in the C*ypdA* strain (Figure 2B, Supplementary Table 2) and were significantly upregulated in the C*ypdA*-*srrA*Δ strain but not in the wild-type, C*ypdA*-*sskA*Δ, or C*ypdA*-*sskA*Δ*srrA*Δ (Figure 2B, Supplementary Table 2), suggesting that downregulation of *ypdA* activated the HogA pathway.

### Downregulation of *ypdA* Causes an Increase in Total and Phosphorylated HogA and Overexpression of *hogA*

**Figure 3:**
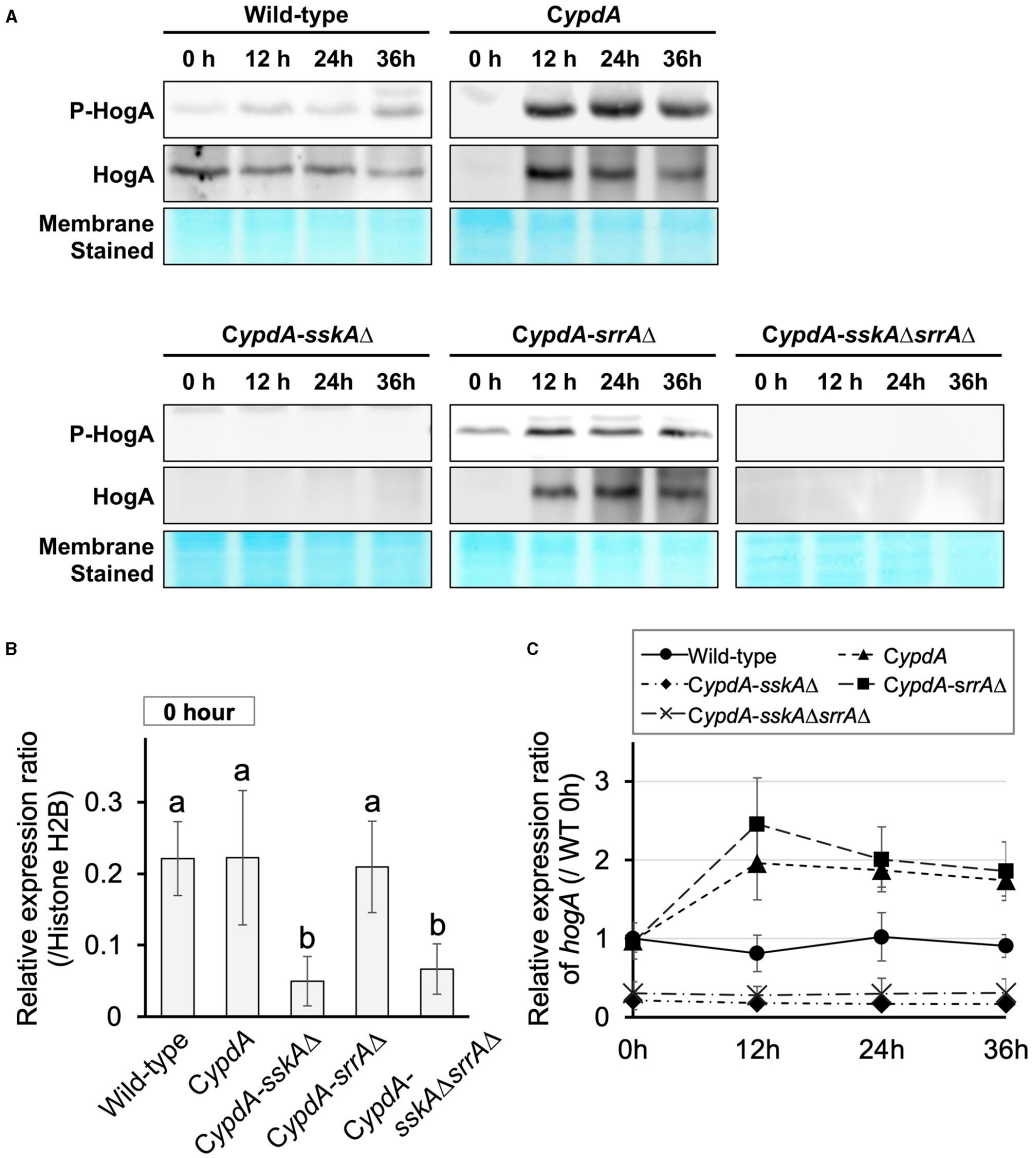
Phosphorylation of HogA protein **(A)** and gene expression of *hogA* **(B,C)** in *Aspergillus nidulans* wild-type, C*ypdA*, and C*ypdA*-derivative strains. **(A)** Western blot analysis of phospho-HogA. HogA protein and stained membrane for confirmation of protein transfer are shown. Samples were prepared from mycelium at 12, 24, and 36 h after transfer from *ypdA*-inducing medium (CDTFY; 0 h) to *ypdA*-repressing medium (CDY). Each lane was loaded with 15 μg protein. **(B)** Quantitative real-time PCR analysis. Conidia (final concentration, 5 × 10^5^ conidia/ml) were cultured in *ypdA*-inducing CDTFY for 20 h before transfer to *ypdA*-repressing CDY medium, at 0 h. Transcript levels of *hogA* were quantified by RT-PCR. Each value represents the expression ratio relative to that of the gene encoding histone H2B in each strain. Error bars represent standard deviations from three independent replicates. Different letters indicate significant differences by Tukey–Kramer's test (*P* < 0.05). **(C)** Time course of *hogA* expression after transfer from *ypdA*-inducing medium (CDTFY; 0 h) to *ypdA*-repressing medium (CDY). Samples were prepared from mycelia at 12, 24, and 36 h, and expression of *hogA* was quantified by RT-PCR. The expression level of the *hogA* gene in each sample was normalized to that of the histone H2B gene (internal control) in the same sample. Each value represents the *hogA* expression ratio relative to expression in the wild-type strain cultured in CDTFY medium for 20 h (WT 0 h). Error bars represent standard deviations from three independent replicates.

To determine whether HogA was activated (i.e., phosphorylated) under *ypdA*-repressing conditions, we performed western blot analysis to detect HogA phosphorylation. HogA was weakly phosphorylated in wild-type cells after transfer to *ypdA*-repressing (CDY) medium (Figure 3A). In C*ypdA* and C*ypdA*-*srrA*Δ cells, HogA was strongly expressed and phosphorylated after transfer to *ypdA-*repressing medium (Figure 3A). In contrast, in C*ypdA*-*sskA*Δ and C*ypdA*-*sskA*Δ*srrA*Δ cells, HogA was scarcely expressed or phosphorylated after transfer to *ypdA*-repressing medium. These results suggested that *ypdA* downregulation caused hyper-expression and phosphorylation of HogA in an SskA–dependent manner. The dependence of HogA expression on SskA signaling was also supported by transcription levels of *hogA* (Figures 3B,C). During vegetative growth, no significant differences were detected in the relative expression ratio of *hogA* among the wild-type, C*ypdA*, and C*ypdA*-*srrA*Δ strains, whereas the levels were significantly lower in the C*ypdA*-*sskA*Δ and C*ypdA*-*sskA*Δ*srrA*Δ strains (Figure 3B). After transfer from *ypdA*-inducing (CDTFY) to *ypdA*-repressing medium, transcription levels of *hogA* were nearly constant in the wild-type strain but were increased significantly in the C*ypdA* and C*ypdA*-*srrA*Δ strains (Figure 3C, Supplementary Table 3). On both media types, transcription levels of *hogA* were consistently lower in C*ypdA*-*sskA*Δ and C*ypdA*-*sskA*Δ*srrA*Δ cells than in the wild-type strain (Figure 3C, Supplementary Table 3).

### Cell Defects Caused by *ypdA* Downregulation Depend on Both SskA and SrrA Pathways

**Figure 4:**
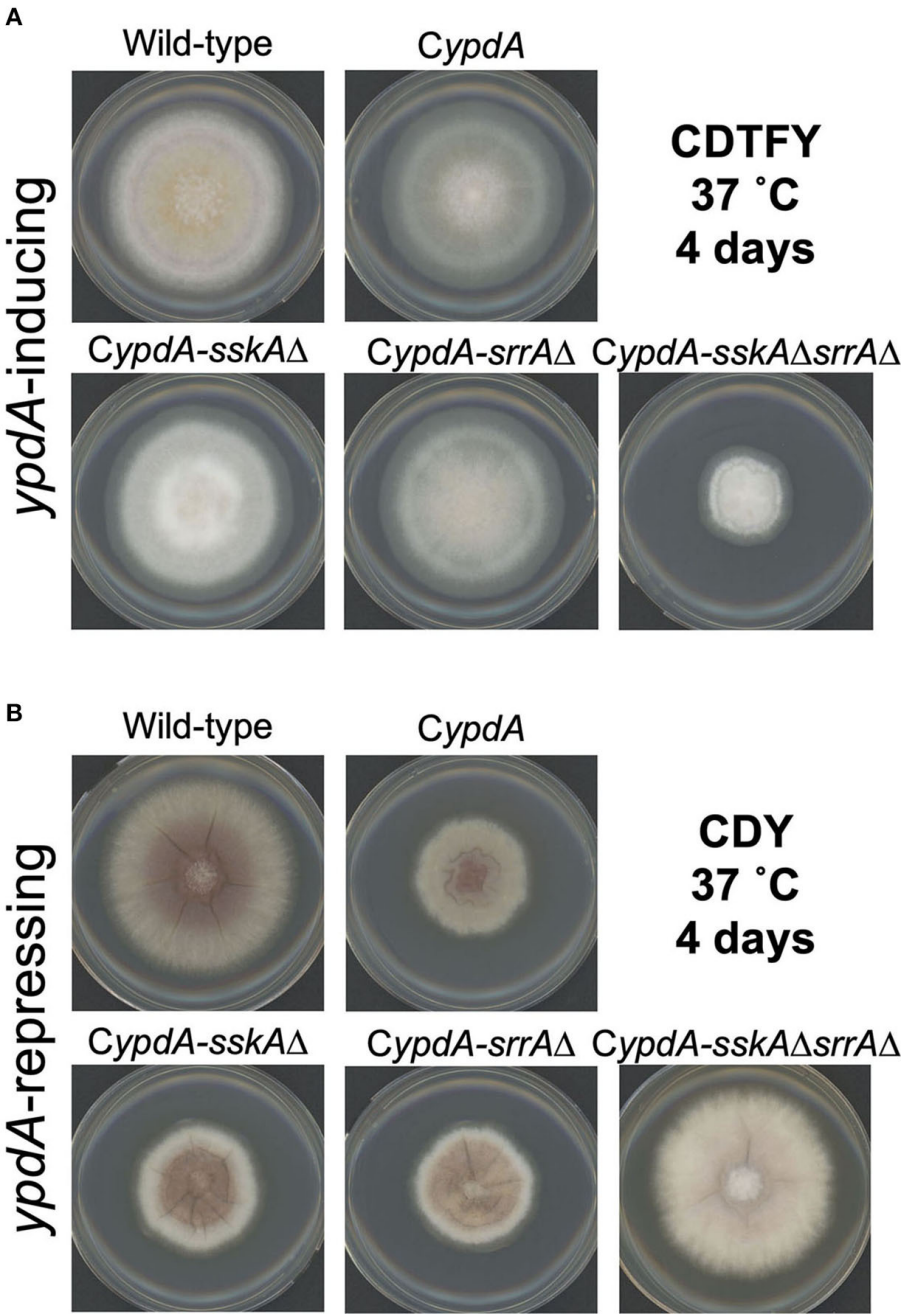
Mycelial growth of *Aspergillus nidulans* wild-type, C*ypdA*, and its derivative strains on *ypdA*-inducing and -repressing plates. Conidia (1 × 10^4^) of each strain were inoculated at the center of agar plates with *ypdA*-inducing CDTFY medium **(A)** or *ypdA*-repressing CDY medium **(B)** and incubated at 37°C for 4 days.

On *ypdA*-inducing (CDTFY) agar plates, no significant differences in colony growth were detected among the wild-type, C*ypdA*, C*ypdA*-*sskA*Δ, and C*ypdA*-*srrA*Δ strains, but C*ypdA*-*sskA*Δ*srrA*Δ strain showed severe growth retardation (Figure 4A, Supplementary Table 4). On *ypdA*-repressing (CDY) agar plates, colonies of the C*ypdA*, C*ypdA*-*sskA*Δ, and C*ypdA*-*srrA*Δ strains were significantly smaller than that of the wild-type strain, but growth of the C*ypdA*-*sskA*Δ*srrA*Δ strain was almost completely restored (Figure 4B, Supplementary Table 4), suggesting that the growth defects caused by *ypdA* downregulation depend on both SskA and SrrA. Although conidial formation is affected in the *sskA*Δ and s*rrA*Δ strains (Hagiwara et al., 2007a), no difference in conidial formation was observed between the wild-type and C*ypdA* strains, regardless of whether they were grown on *ypdA*-inducing or -repressing medium (Supplementary Figure 5A). No effect of *ypdA* repression was observed on conidial germination in the wild-type or C*ypdA* strain (Supplementary Figure 5B). We also tested the growth responses to osmotic stress in the wild-type, C*ypdA*, and its derivative strains together with the *sskA*Δ*srrA*Δ strain (Supplementary Figure 6). Consistent with a previous report (Vargas-Perez et al., 2007), both RRs were required for proper responses to osmotic stress, whether *ypdA* is conditional or not.

**Figure 5:**
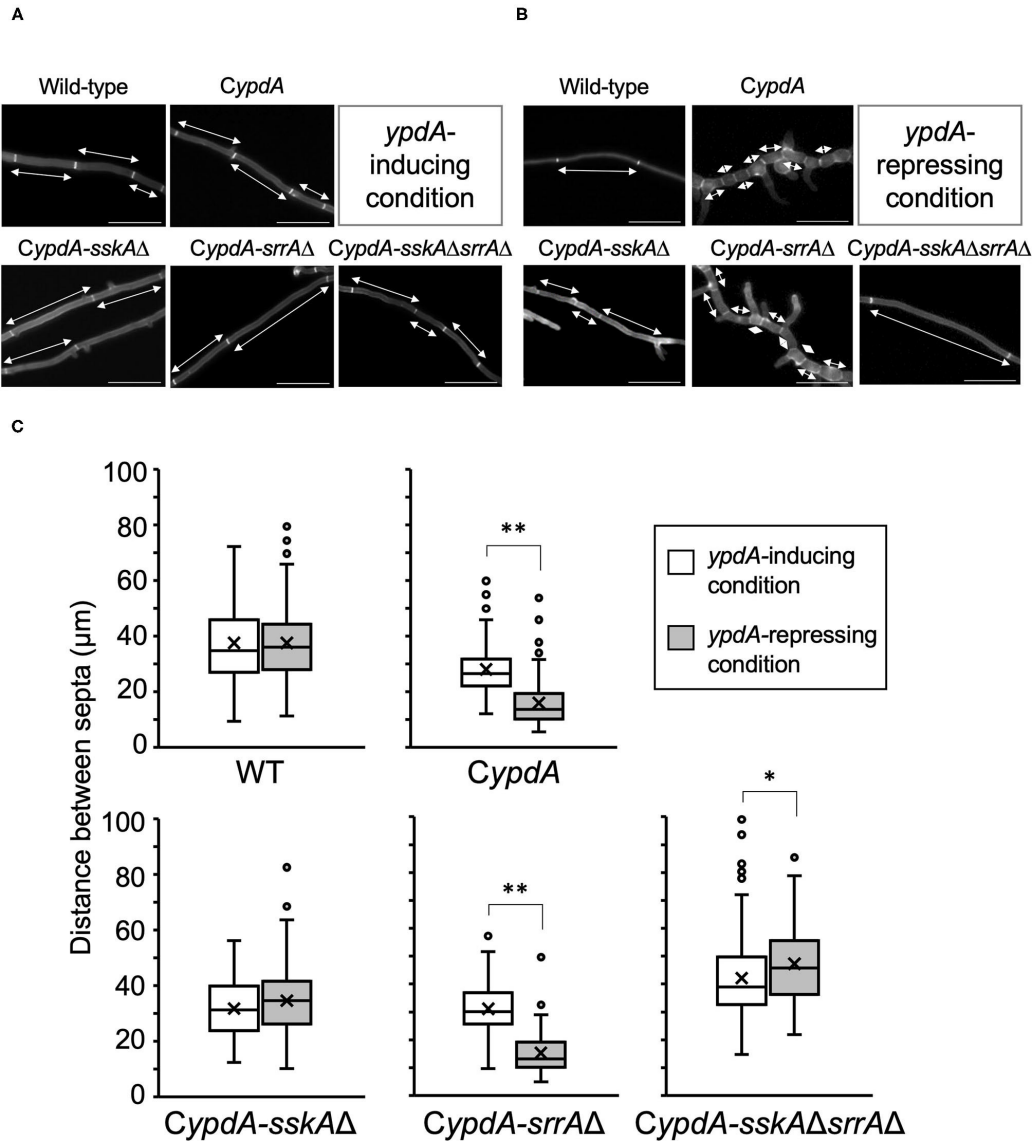
Hyphal morphology **(A,B)** and distance between septa **(C)** of the wild-type, C*ypdA*, and its derivative strains under *ypdA*-inducing and -repressing conditions. Each strain was cultured in liquid *ypdA*-inducing CDTFY **(A)** or *ypdA*-repressing CDY **(B)** medium and stained with Calcofluor White. Double-headed arrows indicate distance between septa. Scale bars, 35 μm. **(C)** White and gray boxes indicate the distribution of distances between septa under the *ypdA*-inducing and *ypdA*-repressing conditions, respectively. Lines in boxes indicate medians, and crosses indicate averages. White circles indicate outliers. Error bars represent the range of maximum and minimum values excluding the outliers. *P < 0.05, **P < 0.01; Welch's *t*-test.

Next, we performed detailed microscopic analysis of the growth defects of C*ypdA*, C*ypdA*-*sskA*Δ, C*ypdA*-*srrA*Δ, and C*ypdA*-*sskA*Δ*srrA*Δ (Figures 5A,B). We compared the average lengths of hyphal cells and distance between septa among the wild-type, C*ypdA*, and C*ypdA*-derivative strains (*n* = 100) (Figure 5C, Supplementary Table 5). Average lengths of hyphal cells were significantly shorter after transfer to *ypdA*-repressing medium than those before the transfer in C*ypdA* and C*ypdA*-*srrA*Δ, but was not significantly altered in the wild-type and C*ypdA*-*sskA*Δ strains (Figure 5C). Hyphal cell length was the same or slightly higher in C*ypdA*-*sskA*Δ*srrA*Δ (Figure 5C, Supplementary Table 5). In addition to being shorter, cells of the C*ypdA* and C*ypdA*-*srrA*Δ strains were balloon-shaped under *ypdA*-repressing conditions, bulging outward between septa (Figure 5B).

### Disorders of Nuclear Distribution Caused by *ypdA* Downregulation

**Figure 6:**
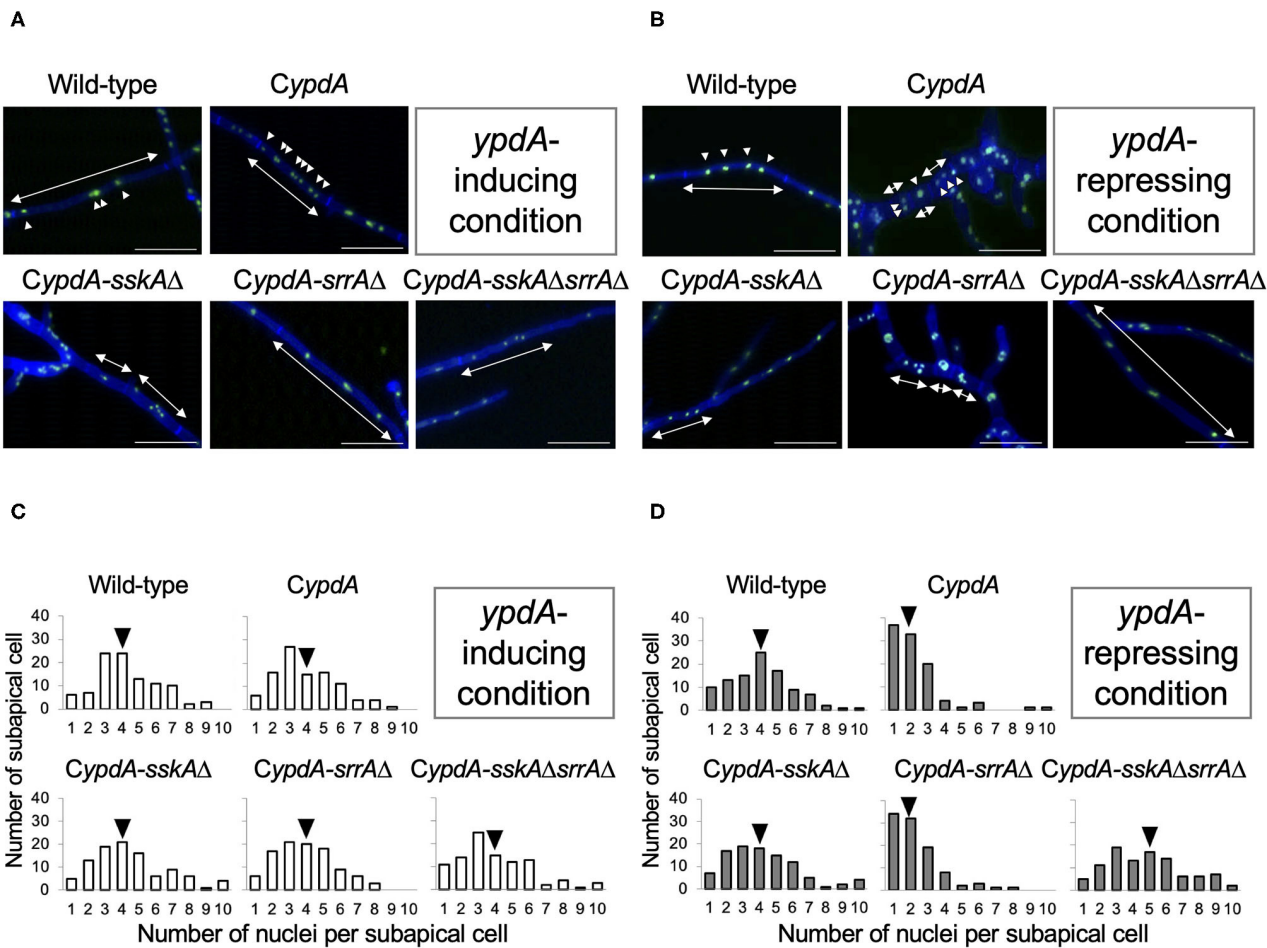
Fluorescent observation of nuclei **(A,B)** and distribution of nuclei between septa **(C,D)** in the wild-type, C*ypdA*, and its derivative strains under *ypdA*-inducing and -repressing conditions. **(A,B)** Nuclei were visualized by using GFP-fused histone H2B under *ypdA*-inducing **(A)** and -repressing **(B)** conditions. Double-headed arrows indicate distance between septa. Arrowheads indicate nuclei. Scale bars, 35 μm. **(C,D)** Nuclei between septa were counted in 100 cells in each strain under *ypdA*-inducing **(C)** and -repressing **(D)** conditions. Arrowheads indicate the median.

Because the average length of hyphal cells of C*ypdA* and C*ypdA*-*srrA*Δ was significantly shorter under *ypdA* downregulation, we examined the distribution of nuclei between septa in the wild-type, C*ypdA*, and C*ypdA*-derivatives under the *ypdA*-inducing and -repressing conditions by using an enhanced green fluorescent protein fused histone H2B (H2B-EGFP) expressing strains (Supplementary Figure 7, Figure 6). The number of nuclei between septa was similar in the wild-type and C*ypdA*-*sskA*Δ strains regardless of culture conditions (Figure 6). In contrast, the number of nuclei between septa of hyphae grown after transfer to the repressing medium was significantly lower than before the transfer in the C*ypdA* and C*ypdA*-*srrA*Δ strains (Figure 6, Supplementary Table 6). In these two strains, the median number of nuclei between septa decreased (from 4 to 2 in both strains) (Figures 6C,D, Supplementary Table 6). In the C*ypdA*-*sskA*Δ*srrA*Δ strain, the nuclear number of hyphae grown after transfer to the repressing medium was slightly greater than before the transfer (Figures 6C,D, Supplementary Table 6). These results suggest that the decrease in nuclear number caused by *ypdA* downregulation is SskA-dependent.

**Figure 7:**
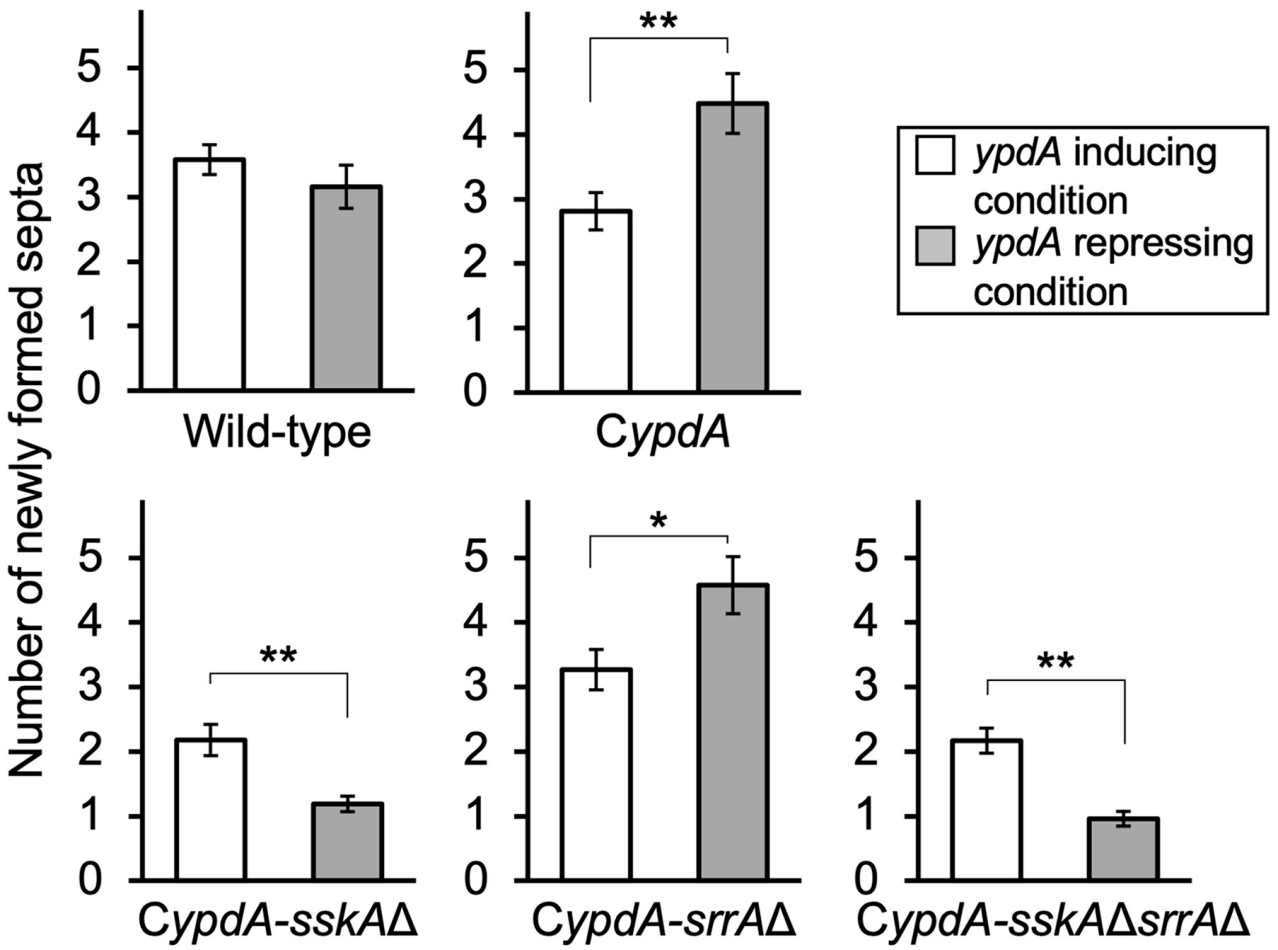
Number of newly formed septa after medium transfer in the wild-type and C*ypdA* strains. The number of newly formed septa in mycelia germinated from a single conidium was calculated by subtracting the average number of septa produced during preculture (CDTFY at 37°C for 10 h, *ypdA*-inducing) from that of septa counted after incubation for 4 h after transfer to *ypdA*-inducing or -repressing medium (*n* = 100 mycelia for each strain in each medium). Error bars represent the standard errors of the mean. *P < 0.05, **P < 0.01; Welch's *t*-test.

To clarify how the timing of septal formation is related to the reduction in nuclear number, we analyzed the number of septa per germling newly formed in 4 h on either *ypdA*-inducing or -repressing medium after transfer from *ypdA*-inducing medium (*n* = 100) (Figure 7, Supplementary Table 7). In the wild-type strain, the number of septa formed after transfer to either medium was not significantly different. In contrast, in C*ypdA* and C*ypdA*-*srrA*Δ the number of septa formed after transfer to *ypdA*-repressing medium was significantly higher than that after transfer to *ypdA*-inducing medium, and in C*ypdA-sskA*Δ and C*ypdA-sskA*Δ*srrA*Δ the number formed after transfer to *ypdA*-repressing medium was significantly lower than that after transfer to *ypdA*-inducing medium (Figure 7). These observations suggest that the decreases in hyphal length and number of nuclei between septa are caused by accelerated septum formation in response to *ypdA* downregulation.

### Fludioxonil Has the Same Effects as *ypdA* Downregulation

**Figure 8:**
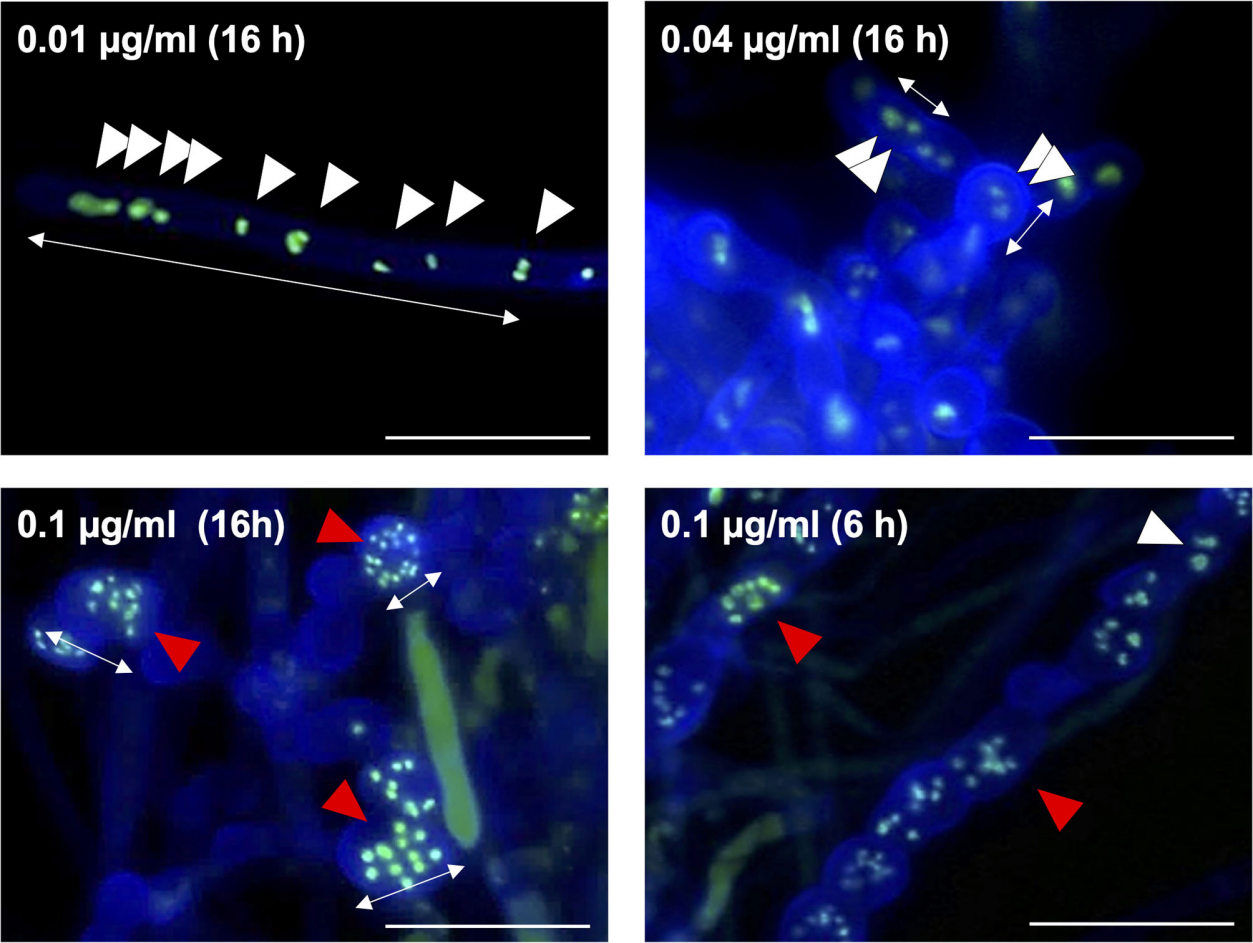
Morphology of hyphae and nuclei after treatment with fludioxonil in the wild-type strain. Conidia (1 × 10^7^ cells) were incubated in liquid CDTFY medium (67 ml) for 20 h at 37°C. Mycelia were transferred to CDTFY medium with the indicated concentrations of fludioxonil and incubated for 6 or 16 h at 37°C. Lines with arrows indicate distance between septa. White arrowheads indicate nuclei. Red arrowheads indicate fragmented nuclei. Scale bars, 35 μm.

**Figure 9:**
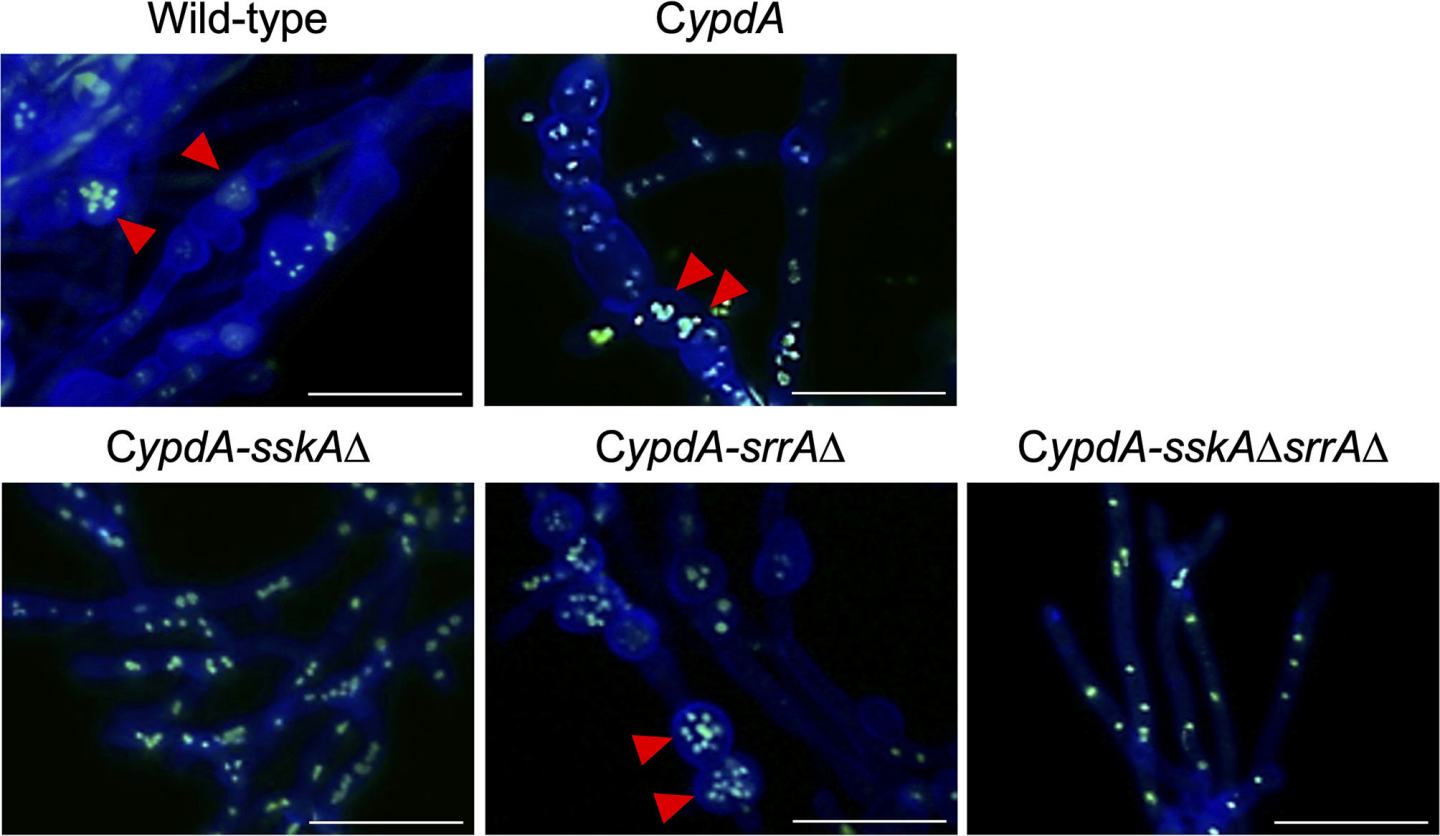
Morphology of hyphae and nuclei under *ypdA*-inducing + 0.1 μg/ml fludioxonil conditions in the wild-type and C*ypdA* strains. Strains were cultured for 20 h in liquid CDTFY (*ypdA*-inducing) medium at 37°C, then transferred to CDTFY medium with fludioxonil (0.1 μg/ml) and incubated for 16 h at 37°C. Red arrowheads indicate fragmented nuclei. Bars, 35 μm.

Fludioxonil is an inhibitor of group III HKs (Catlett et al., 2003) in fungi; the HAMP domain is thought to be the target of fludioxonil (Ochiai et al., 2001, Yoshimi et al., 2004). Fludioxonil treatment of *A. nidulans* wild-type strains leads to activation of the HOG MAP kinase cascade *via* inhibition of the HK NikA. Therefore, fludioxonil treatment leads to constitutive activation of HogA as well as *ypdA* downregulation in *A. nidulans* (Lara-Rojas et al., 2011; Figure 3). When wild-type cells were exposed to fludioxonil, they showed changes similar to those caused by *ypdA* repression, depending on the fludioxonil concentration (Figures 6B, 8). No obvious changes in cell length and number of nuclei between septa were observed after treatment with 0.01 μg/ml fludioxonil for 16 h, but cells became shorter, with a balloon-like shape and fewer nuclei between septa, after treatment with 0.04 μg/ml fludioxonil for 16 h (Figure 8, Supplementary Table 8). Balloon-shaped cells and fragmented nuclei were observed in some cells after treatment with 0.1 μg/ml fludioxonil for 6 or 16 h (Figure 8, Supplementary Table 8). Treatment with 0.1 μg/ml fludioxonil increased the percentage of cells without nuclei (Supplementary Table 8). In the C*ypdA* and C*ypdA-srrA*Δ strains, treatment with 0.1 μg/ml fludioxonil for 16 h reduced the number of nuclei between septa and fragmented nuclei, but the percentage of cells with fragmented nuclei was reduced in the C*ypdA-sskA*Δ and C*ypdA-sskA*Δ*srrA*Δ strains (Figure 9, Supplementary Table 9). At 16 h after transfer to *ypdA*-repressing medium, the C*ypdA* strain showed reduced numbers of nuclei and fragmented nuclei (Supplementary Figure 8, Supplementary Table 10). This phenomenon was also observed in the C*ypdA*-*srrA* strain under *ypdA* repression, but no morphological changes in nuclei were observed in the C*ypdA-sskA*Δ and C*ypdA-sskA*Δ*srrA*Δ strains (Supplementary Figure 8, Supplementary Table 10). The C*ypdA-sskA*Δ strain showed reduced septum formation compared to the C*ypdA-srrA*Δ strain in the presence of fludioxonil (Supplementary Figure 9) or *ypdA* repression (Figure 7). These observations suggest that fludioxonil treatment produced the same effects as *ypdA* downregulation, in an SskA–dependent manner. As in the case of the *sskA*Δ*srrA*Δ strain, the C*ypdA-sskA*Δ*srrA*Δ strain grew vigorously even in the presence of fludioxonil (Supplementary Figure 10), which is consistent with the restoration of its growth under *ypdA*-repressing conditions (Figure 4).

### Vacuole Development Caused by *ypdA* Downregulation and Fludioxonil Treatment

**Figure 10:**
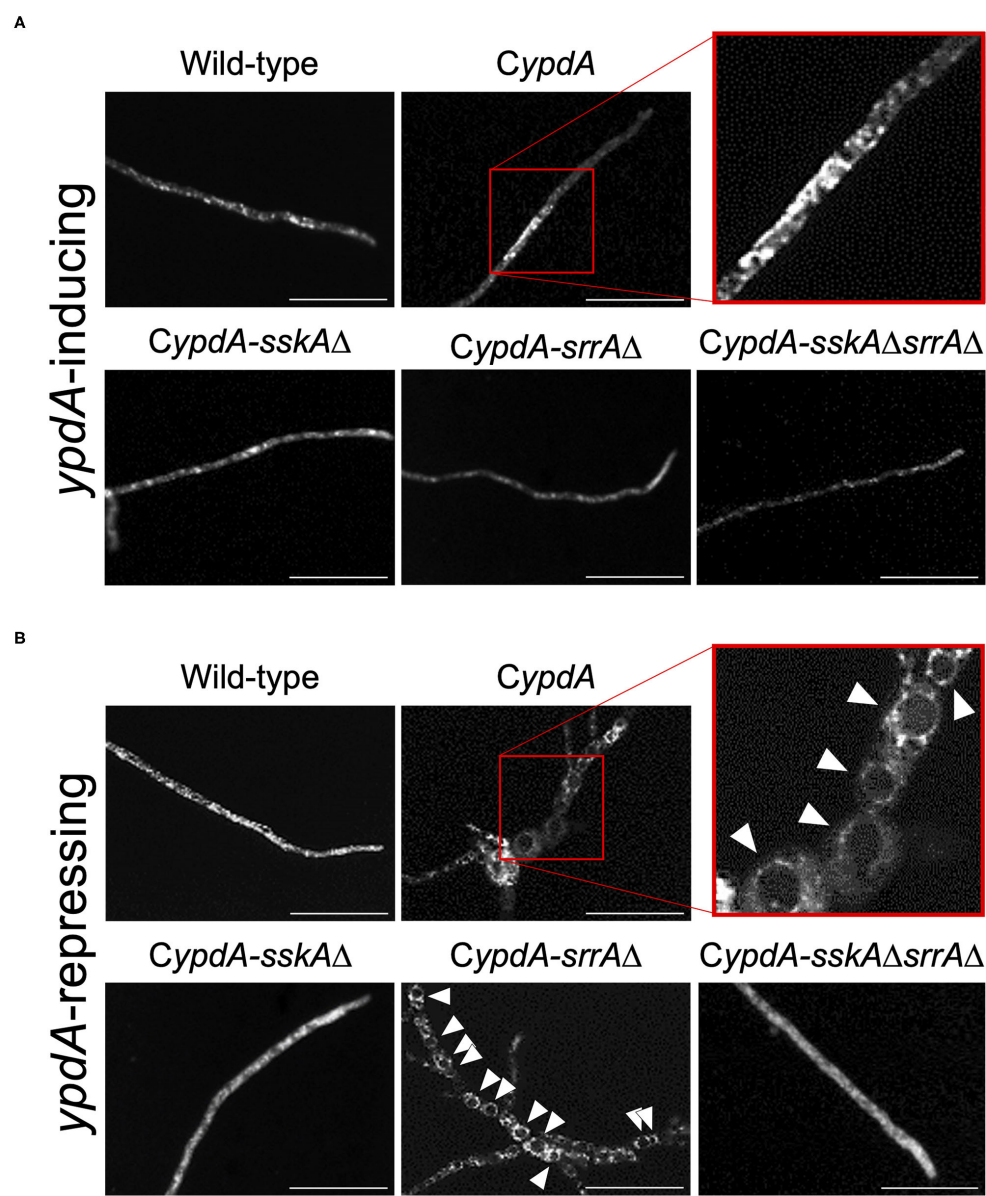
Vacuole morphology of the wild-type and C*ypdA* strains under *ypdA*-inducing and -repressing conditions. Conidia of each strain were cultured in CDTFY (*ypdA*-inducing) medium for 8 h at 37°C, then transferred to CDTFY (**A**, *ypdA*-inducing) or CDY (**B**, *ypdA*-repressing) conditions and incubated for 4 h at 37°C. After incubation, mycelia were stained with FM4-64 and vacuoles were observed by fluorescence microscopy. Arrowheads indicate vacuoles. Bars, 35 μm.

**Figure 11:**
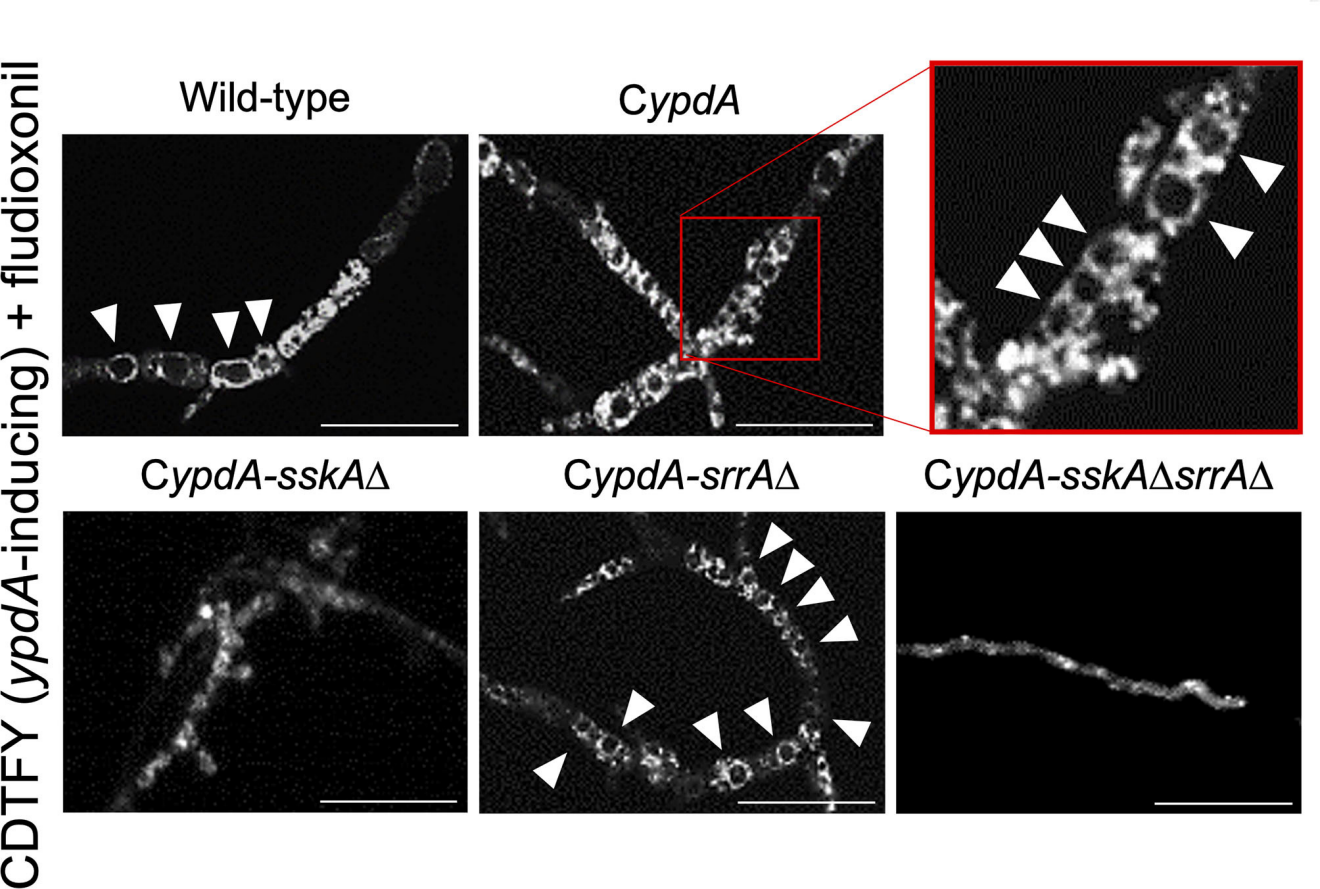
Vacuole morphology of the wild-type and C*ypdA* strains treated with fludioxonil under *ypdA*-inducing conditions. Conidia of each strain were cultured in CDTFY (*ypdA*-inducing) medium for 8 h at 37°C, then transferred to CDTFY with 0.1 μg/ml fludioxonil and incubated for 4 h at 37°C. After incubation, mycelia were stained with FM4-64 and vacuoles were observed by fluorescence microscopy. Arrowheads indicate vacuoles. Bars, 35 μm.

Since *ypdA* downregulation increased the ratio of cells with reduced numbers of nuclei in the C*ypdA* and C*ypdA-srrA*Δ strains, we microscopically observed the intracellular membranes by FM4-64 staining. All C*ypdA* derivatives and the wild-type strain showed normal size of vacuoles under *ypdA*-inducing conditions (Figure 10A). Downregulation of *ypdA* transcription resulted in formation of many large vacuoles in the C*ypdA* and C*ypdA-srrA*Δ strains, but not in the C*ypdA-sskA*Δ or C*ypdA-sskA*Δ*srrA*Δ strains (Figure 10B). We also examined whether fludioxonil treatment of *A. nidulans* induces formation of large vacuoles (Figure 11). Under *ypdA* induction, fludioxonil treatment led to formation of many large vacuoles in the wild-type, C*ypdA*, and C*ypdA-srrA*Δ strains but not in the C*ypdA-sskA*Δ and C*ypdA-sskA*Δ*srrA*Δ strains (Figure 11). These results suggest that fludioxonil treatment and *ypdA* downregulation increased the formation of large vacuoles in a HOG pathway–dependent manner.

**Figure 12:**
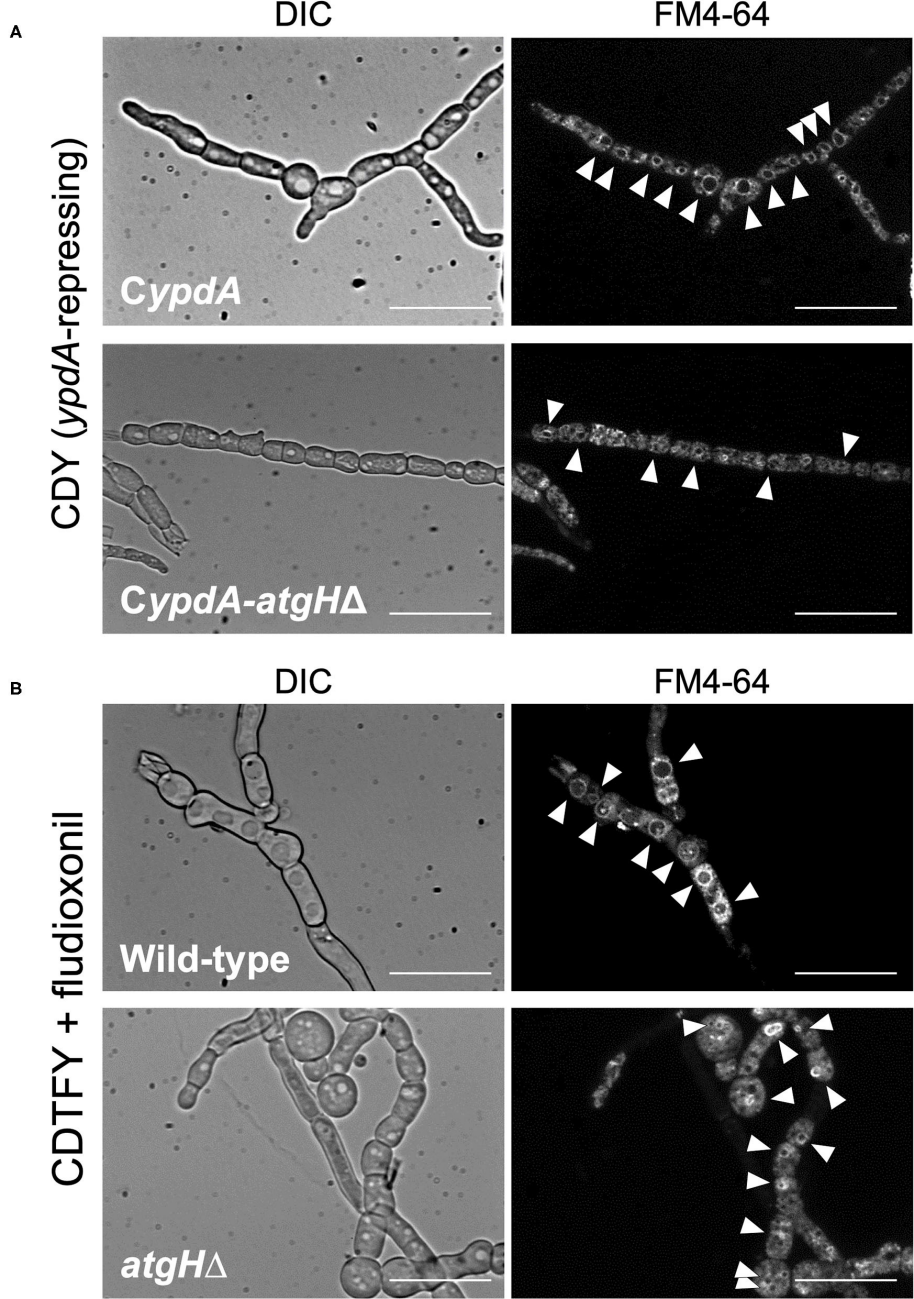
Vacuole morphology of *atgH*-disrupted strains derived from wild-type and C*ypdA* strains under the *ypdA*-repressing condition and fludioxonil treatment. **(A)** Conidia of the C*ypdA* and C*ypdA*-*atgH*Δ strains were cultured in CDTFY (*ypdA*-inducing) medium for 8 h at 37°C. Mycelia were transferred to CDY medium (*ypdA*-repressing) and incubated for 4 h at 37°C. Mycelia were stained with FM4-64 and vacuoles were observed by fluorescence microscopy. **(B)** Conidia of the wild-type and *atgH*Δ strains were cultured in CDTFY medium for 8 h at 37°C. Mycelia were transferred to CDTFY with 0.1 μg/ml fludioxonil and incubated for 4 h at 37°C. Mycelia were stained with FM4-64 and vacuoles were observed by fluorescence microscopy. Arrowheads indicate vacuoles. Bars, 35 μm.

To investigate whether vacuole development and reduction in the number of nuclei were related to autophagy, we constructed strains with the deletion of *atgH*, which encodes an autophagy-related ubiquitin-like protein (Shpilka et al., 2011), using the wild-type and C*ypdA* strains (*atgH*Δ and C*ypdA-atgH*Δ, respectively; Supplementary Figure 11). Under *ypdA* repression, the C*ypdA-atgH*Δ strain showed fewer, smaller vacuoles than the C*ypdA* strain, although cells of the C*ypdA-atgH*Δ strain were balloon-like (Figure 12A, Supplementary Table 11). Cell shape remained abnormal, but *atgH* disruption also abrogated formation of the large vacuoles induced by fludioxonil treatment (Figure 12B, Supplementary Table 11). In addition, no significant differences were observed in fludioxonil sensitivity in the wild-type and *atgH*Δ strains (Supplementary Figure 12). These results suggest that the abnormal development of vacuoles is not directly related to the cell defects induced by fludioxonil treatment and *ypdA*-downregulation.

## Discussion

In this study, we demonstrated that downregulation of *ypdA* transcription causes severe growth defects and abnormal hyphal morphology in *A. nidulans*. These results support the notion that *ypdA* is an essential gene, although the molecular mechanism(s) underlying the lethality caused by downregulation of *ypdA* remain(s) unclear. In the present study, we significantly downregulated intracellular levels of YpdA protein by downregulating *ypdA* transcription in C*ypdA* and its derivatives C*ypdA*-*sskA*Δ, C*ypdA*-*srrA*Δ, and C*ypdA*-*sskA*Δ*srrA*Δ (Figure 1). Loss of YpdA increased the levels of total and phosphorylated HogA in the C*ypdA* and C*ypdA*-*srrA*Δ strains (Figure 3A), which reflected hyper-activation of HOG MAPK signaling. The activation of HOG MAPK signaling was maintained for at least 36 h, and the transcription of the HOG pathway–regulated gene *catA* (Figure 2) and *hogA* itself (Figures 3B,C) were persistently upregulated. The increased level of *hogA* transcript reflected the high levels of HogA protein (Figure 3A). Taking into consideration that the *hogA* expression level was lower in the C*ypdA*-*sskA*Δ and C*ypdA*-*sskA*Δ*srrA*Δ strains in *ypdA*-repressing medium (CDY), these results suggested that the transcription of *hogA* was regulated by the HOG pathway *via* autoregulation. Although autoregulation of MAP kinase gene expression has been reported in the cell wall integrity signaling pathway in *A. nidulans* (Fujioka et al., 2007), this is the first clear demonstration of the autoregulation of the HOG pathway in filamentous fungi.

The growth defects caused by *ypdA* downregulation were restored by double disruption of the SskA and SrrA pathways, but not by disruption of either pathway alone (Figure 4B). This result strongly suggests that the lethality caused by downregulation of *ypdA* is not dependent on either the SskA or SrrA pathway alone, but on both pathways. In this respect, our findings differ from those in *S. cerevisiae* (Posas et al., 1996). Downregulation of *S. cerevisiae YPD1* results in lethal, constitutive activation of the Hog1 MAPK cascade, which is almost completely suppressed by deletion of *SSK1, HOG1*, or *PBS2* (Posas et al., 1996). Consequently, the lethality caused by downregulation of *YPD1* expression fully depends on the Hog1 MAPK cascade in *S. cerevisiae*. Interestingly, even under *ypdA*-inducing conditions, severe growth retardation was observed only in the C*ypdA*-*sskA*Δ*srrA*Δ strain (Figure 4A). Thus, the activation of one response regulator appears to be sufficient for the phosphate group on YpdA to be processed at normal levels. However, deletion of both response regulators may hamper phosphate group transfer, leading to excessive accumulation of phosphorylated YpdA and to severe growth retardation in the C*ypdA*-*sskA*Δ*srrA*Δ strain. Further study is needed to elucidate the relationship between over-accumulation of phosphorylated YpdA and cell toxicity.

In filamentous fungi including aspergilli, *N. crassa*, and most plant pathogens, fungicides such as fludioxonil and iprodione are thought to target the His-Asp phosphorelay system (Ochiai et al., 2001; Yoshimi et al., 2004; Motoyama et al., 2005; Viaud et al., 2006; Hagiwara et al., 2007b). The growth-inhibitory effects of the fungicides have been shown to depend on group III HKs, deletion of which confers resistance. In *A. nidulans, nikA* encodes a group III HK, and we previously showed that a *nikA* deletion mutant is resistant to fludioxonil (Hagiwara et al., 2007b). The *sskA*Δ*srrA*Δ and *hogA*Δ*srrA*Δ double mutants are also resistant to fludioxonil (Hagiwara et al., 2007b). The growth defects caused by fludioxonil treatment or by *ypdA* downregulation are both restored by disruption of both the SskA and SrrA pathways, in a similar manner. These observations indicate that the growth inhibitory-effects of the fungicide and *ypdA* downregulation may be caused by disruption of His-Asp phosphorelay signaling, in which signals are transmitted from NikA to RRs (SskA and SrrA). In fact, the HogA MAPK cascade is abnormally activated by fludioxonil treatment (Furukawa et al., 2007) and *ypdA* downregulation (Figure 3A), both of which depends on SskA pathways (Hagiwara et al., 2007a, 2009; Figure 3A).

The abnormal cell morphology, with shorter, balloon-shaped cells caused by YpdA repression, was observed in the C*ypdA* and C*ypdA*-*srrA*Δ strains (Figure 5B). However, in the C*ypdA-sskA*Δ and C*ypdA-sskA*Δ*srrA*Δ strains, in which *sskA* was disrupted, cell length was restored to that of the wild-type strain (Figure 5), indicating that the abnormalities in cell morphology are dependent on the SskA pathway. This suggested that septum formation was regulated by a pathway downstream of SskA, for example, the HogA pathway. Phenotypes similar to the septum defects observed in the C*ypdA*Δ and C*ypdA*-*srrA*Δ strains have been reported in a study of the two-component system in the fission yeast *Schizosaccharomyces pombe* (Shieh et al., 1997; Shiozaki et al., 1997). Mcs4, an ortholog of SskA in *A. nidulans*, regulates mitotic initiation in a manner dependent on Sty1, an ortholog of HogA in *A. nidulans*. Cells of the *mcs4*Δ mutant are longer than those of the wild-type strain, a result of the delay in septum formation caused by G2 arrest, which prevents transition to the M phase. Conversely, deletion of *spy1*, which encodes an ortholog of YpdA, can grow but leads to reduced cell length, caused by enhanced transition to M phase. However, in the *mcs4*Δ*spy1*Δ strain, cell length is restored to that of the *mcs4*Δ strain (Aoyama et al., 2000). Given the findings in *S. pombe* and the results of the present study, HogA may also regulate the transcription of cell cycle–related genes *via* its target transcription factor in *A. nidulans*.

The number of nuclei per cell was significantly decreased in the C*ypdA* and C*ypdA*-*srrA*Δ strains under the *ypdA*-repressing condition (Figures 6C,D). In the C*ypdA-sskA*Δ and C*ypdA-sskA*Δ*srrA*Δ strains, the number of nuclei per cell was restored to that of the wild-type strain (Figures 6C,D), indicating that nuclear distribution in cells may be dependent on the SskA pathway. This suggested that nuclear distribution, as well as septum formation, is regulated by a pathway downstream of SskA. When hyphal cells of the wild-type strain were treated with 0.1 μg/ml fludioxonil for 16 h, nuclear fragmentation was observed (Figure 8). When the fludioxonil concentration or treatment time was reduced, nuclear fragmentation was suppressed and the number of nuclei per cell tended to decrease in the wild-type strain (Figure 8). In addition, when hyphal cells of the C*ypdA* and C*ypdA-srrA*Δ strains were treated with 0.1 μg/ml fludioxonil for 16 h under the *ypdA*-inducing condition, cells with reduced numbers of nuclei and fragmented nuclei were observed (Figure 9). Abnormal nuclear morphology was not observed in cells of the C*ypdA-sskA*Δ and C*ypdA-sskA*Δ*srrA*Δ strains under the *ypdA*-inducing condition, indicating that the abnormal nuclear morphology is dependent on the SskA pathway.

Comparison of the cellular alterations caused by fludioxonil and *ypdA* repression showed that abnormal cells with reduced and fragmented nuclei were present in the C*ypdA* and C*ypdA-srrA*Δ strains under both conditions (Figure 8, Supplementary Figure 8). However, no morphological changes in nuclei were observed in the C*ypdA-sskA*Δ and C*ypdA-sskA*Δ*srrA*Δ strains under the *ypdA*-repressing condition (Supplementary Figure 8). Thus, fragmentation and decreased numbers of nuclei are common to the *ypdA*-repressing and fludioxonil treatments, in an SskA pathway–dependent manner.

The number of newly formed septa was not significantly different in the wild-type strain at 4 h after transfer to the *ypdA*-repressing condition, compared to the inducing condition (Figure 7). However, the C*ypdA* and C*ypdA-srrA*Δ strains showed an increased number of septa after transfer to the *ypdA*-repressing condition (Figure 7). This result suggested that the decreases in cell length between septa and in nuclear number, observed in the *ypdA*-repressing condition, were caused by enhanced septum formation. The C*ypdA-sskA*Δ and C*ypdA-sskA*Δ*srrA*Δ strains showed reduced septum formation in a certain period (4 h) compared to the C*ypdA-srrA*Δ strain in response to *ypdA* repression (Figure 7), which suggests delayed formation of each septum in the strains. The delayed septum formation in the C*ypdA-sskA*Δ and C*ypdA-sskA*Δ*srrA*Δ strains suggests that septum formation is regulated in an SskA pathway–dependent manner. In studies on regulators of septum formation in *A. nidulans*, responses such as enhanced and delayed septum formation have been reported. ParA, a regulatory subunit of protein phosphatase 2A in *A. nidulans*, negatively regulates septation by dephosphorylation of septation initiation network proteins. Disruption of *parA* in *A. nidulans* enhances septum formation, and overexpression of *parA* leads to cessation of septum formation (Zhong et al., 2014). MztA was identified by RNA-seq and genetic analysis as a positive regulator of septum formation that acted antagonistically to ParA in *A. nidulans* (Jiang et al., 2018). Septum formation is delayed in the *A. nidulans mztA* disruptant, and the phenotypes of the *parA* and *mztA* disruptants are similar to the phenotypes observed in this study, with either delayed or accelerated septum formation. Since expression of genes involved in the regulation of septum formation, such as *parA* and *mztA*, may be altered in strains with abnormal septum formation under *ypdA*-repressing conditions, the relationship between changes in their expression and YpdA suppression should be clarified in future studies.

In response to fludioxonil, the C*ypdA-sskA*Δ strain showed delayed septum formation compared to C*ypdA-srrA*Δ (Supplementary Figure 9), as it did to *ypdA* repression (Figure 7). On medium containing 0.1 μg/ml fludioxonil, the C*ypdA-sskA*Δ strain was strongly inhibited, but grew at a slightly faster than the C*ypdA* and C*ypdA-srrA*Δ strains, whereas the C*ypdA-sskA*Δ*srrA*Δ grew well (Supplementary Figure 10). Fludioxonil treatment caused growth defects in the C*ypdA-sskA*Δ strain without reducing hyphal length between septa. Thus, nuclear fragmentation may be one of the lethal factors, but there may also be SrrA pathway–dependent lethal factor(s). Although signaling downstream of SrrA is still obscure, YpdA was shown in our study to play a significant role in *A. nidulans* His-Asp phosphorelay signaling with respect to growth and morphology, besides regulation of the HogA pathway.

In the wild-type, C*ypdA*, and C*ypdA*-derivative strains, large vacuoles were not observed under the *ypdA*-inducing condition (Figure 10A). However, large vacuoles developed in the C*ypdA* and C*ypdA-srrA*Δ strains under the *ypdA*-repressing condition (Figure 10B). In response to fludioxonil, large vacuoles also developed in the wild-type, C*ypdA*, and C*ypdA-srrA*Δ strains (Figure 11). Vacuole development was not observed in C*ypdA-sskA*Δ and C*ypdA-sskA*Δ*srrA*Δ, even under the *ypdA*-repressing condition or fludioxonil treatment (Figures 10B, 11). Therefore, vacuole formation, in addition to septum formation and nuclear distribution, also appears to be regulated in an SskA pathway–dependent manner in response to either *ypdA* repression or fludioxonil treatment. In the C*ypdA* and C*ypdA-srrA*Δ strains, in which large vacuoles were observed under the *ypdA*-repressing condition, HogA MAP kinase was abnormally activated (Figure 3A). The HOG-MAP kinase cascade regulates glycerol biosynthetic genes in *A. nidulans* (Fillinger et al., 2001; Han and Prade, 2002). Therefore, we hypothesized that in the C*ypdA* and C*ypdA-srrA*Δ strains glycerol abnormally accumulated in the cytoplasm due to activation of HogA, and that vacuoles developed abnormally in response. This will need to be investigated in future studies.

The development of abnormally large vacuoles observed in the C*ypdA* strain under the *ypdA*-repressing condition was suppressed in the C*ypdA*-*atgH*Δ strain (Figure 12A). In response to fludioxonil, the development of abnormally large vacuoles was observed in the wild-type strain, whereas no vacuole development was observed in the *atgH*Δ strain. This suggests that AtgH may be involved in formation of abnormal vacuoles in response to fludioxonil in the wild-type and under *ypdA* repression in the C*ypdA* strain. Although abnormal vacuole development was suppressed by *atgH* disruption, development of balloon-like cells was not (Figure 12). This indicates that abnormal development of vacuoles is not a direct cause of the balloon-like cells. We hypothesized that abnormal development of vacuoles would enhance autophagy, resulting in autophagic cell death. If this hypothesis is correct, the *atgH*Δ strain, in which vacuoles do not develop abnormally, should be resistant to fludioxonil. However, disruption of *atgH* did not alter fludioxonil sensitivity (Supplementary Figure 12). Thus, abnormal development of vacuoles does not appear to be directly related to the lethality caused by *ypdA*-repressing and fludioxonil treatment.

**Figure 13:**
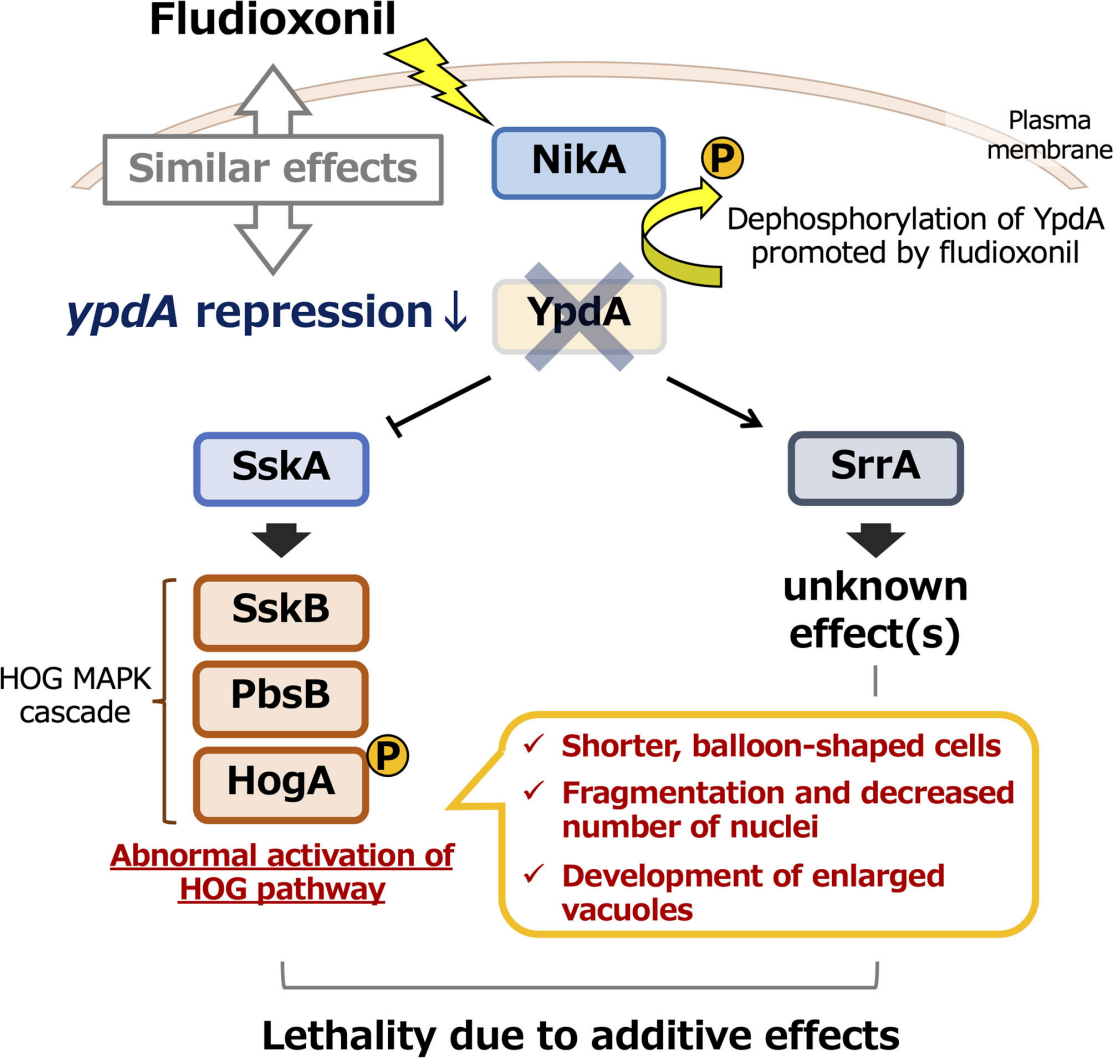
Model of the effects of *ypdA* downregulation and fludioxonil treatment on two-component signaling in *Aspergillus nidulans*. Downregulation of *ypdA* and fludioxonil treatment have similar effects. Although morphological abnormalities depend only on the SskA pathway, the lethality caused by YpdA suppression or fludioxonil treatment depends on both the SskA and SrrA pathways.

Fludioxonil may convert group III HKs into phosphatases (Lawry et al., 2017). In *S. cerevisiae* expressing Drk1, a group III HK from *B. dermatitidis*, Drk1 behaved as a phosphatase rather than as a kinase in response to fludioxonil; it dephosphorylated its downstream target, Ypd1, resulting in constitutive activation of the HOG pathway (Lawry et al., 2017). Our observations of constitutive activation of HOG pathway in response to *ypdA* downregulation or fludioxonil treatment, due to reduced YpdA activity and consequent perturbation of the downstream signaling pathway, are consistent with this report. The conversion of group III HKs into phosphatases by fludioxonil might also help to explain mycelial growth in the C*ypdA-sskA*Δ*srrA*Δ strain on the media containing fludioxonil: in the absence of both response regulators, the phosphate group might be removed from excessively accumulated phosphorylated YpdA by fludioxonil-converted HKs, resulting in vigorous growth of this strain (Supplementary Figure 10). Investigating this hypothesis might be the object of a future study. In addition, the levels of Ypd1 protein are reportedly not affected by fludioxonil treatment (Lawry et al., 2017). Hence, fludioxonil may inhibit growth by causing HK to function as a phosphatase rather than by inhibiting YpdA expression. This would lead to abnormal activation of the HOG pathway, similar to the effects of *ypdA* downregulation. Thus, the YpdA function presented here, the growth-inhibitory effects of fludioxonil, and the lethality of downregulation of *ypdA* may act *via* similar or identical mechanisms, as summarized graphically in Figure 13. Additional studies, including analysis of pathways downstream of SrrA, are needed to elucidate the mechanism underlying the mode of action of phenylpyrrole- or dicarboximide-type fungicides.

## Materials and Methods

### Strains, Media, and Transformation

The fungal strains used in this study are listed in Table 1. The *A. nidulans* ABPU1 (Δ*ligD*) strain was used as the control strain and for all genetic manipulations in the present study. For the *A. nidulans* conditional *ypdA* studies, ABPU1 cells were transformed with the gene-replacement constructs described in the “Construction of gene-replacement cassettes” subsection. Czapek–Dox (CD) medium was used for standard culture (Fujioka et al., 2007). A modified medium, designated CDTFY, CD medium containing 50 mM threonine, 0.2% (w/v) fructose, and 0.2% (w/v) yeast extract instead of 1% (w/v) glucose as a carbon source was used for up-regulation of *alcA* promoter–driven *ypdA* (Ichinomiya et al., 2002). For down-regulation of *alcA* promoter–driven *ypdA*, CD medium containing 2% (w/v) glucose and 0.2% (w/v) yeast extract (designated CDY) was used. Conidia were harvested in PBS + Tween 20 (0.1%, v/v) and counted in a hemacytometer. For mycelial growth tests, conidial suspensions of each strain (1 × 10^4^ conidia) were spotted on the centers of CDTFY or CDY plates and incubated at 37°C for 4 days. For tests of sensitivity to fludioxonil, the same conidial suspensions were spotted on the centers of CDTFY plates containing fludioxonil (0.01, 0.05, 0.1, 0.5, 1.0 μg/ml) and incubated at 37°C for 4 days. The dose response was determined by plotting the mean diameters of the colonies on media with fludioxonil as a percentage of those on control medium (containing 0.1% DMSO as a solvent for fludioxonil). To investigate gene and protein expression, conidia (5 × 10^5^ conidia/ml) were inoculated into liquid CDTFY medium and cultured at 37°C for 20 h. Mycelia were harvested, washed with sterile distilled water, transferred into liquid CDTFY or CDY medium, and cultured for 36 h. Mycelia were harvested at 12, 24, and 36 h. DNA extraction, RNA extraction, reverse transcriptase reactions, and fungal transformations were performed as described previously (Yoshimi et al., 2013).

**Table 1:** Strains used in this study.

**Strains**	**Genotypes**	**References**
ABPU1 (Δ*ligD*)	*biA1, pyrG89, wA3, argB2, pyroA4, veA1, ligD*::*ptrA*	Yoshimi et al., 2013
C*ypdA*	*biA1, pyrG89, wA3, argB2, pyroA4, veA1, ligD*::*ptrA*, P*alcA-ypdA*::*pyrG*	This study
C*ypdA-sskAΔ*	*biA1, pyrG89, wA3, argB2, pyroA4, veA1, ligD*::*ptrA*, P*alcA-ypdA*::*pyrG, sskA*::*argB*	This study
C*ypdA-srrAΔ*	*biA1, pyrG89, wA3, argB2, pyroA4, veA1, ligD*::*ptrA*, P*alcA-ypdA*::*pyrG, srrA*::*argB*	This study
C*ypdA-sskA*Δ*srrAΔ*	*biA1, pyrG89, wA3, argB2, pyroA4, veA1, ligD*::*ptrA*, P*alcA-ypdA*::*pyrG, sskA*::*argB, srrA*::*argB*	This study
ABPU1 *h2b-egfp*	*biA1, pyrG89, wA3, argB2, pyroA4, veA1, ligD*::*ptrA, h2b-egfp*::*pyroA*	This study
C*ypdA h2b-egfp*	*biA1, pyrG89, wA3, argB2, pyroA4, veA1, ligD*::*ptrA*, P*alcA-ypdA*::*pyrG, h2b-egfp*::*pyroA*	This study
C*ypdA-sskAΔ h2b-egfp*	*biA1, pyrG89, wA3, argB2, pyroA4, veA1, ligD*::*ptrA*, P*alcA-ypdA*::*pyrG, sskA*::*argB, h2b-egfp*::*pyroA*	This study
C*ypdA-srrAΔ h2b-egfp*	*biA1, pyrG89, wA3, argB2, pyroA4, veA1, ligD*::*ptrA*, P*alcA-ypdA*::*pyrG, srrA*::*argB, h2b-egfp*::*pyroA*	This study
C*ypdA-sskA*Δ*srrAΔ h2b-egfp*	*biA1, pyrG89, wA3, argB2, pyroA4, veA1, ligD*::*ptrA*, P*alcA-ypdA*::*pyrG, sskA*::*argB, srrA*::*argB, h2b-egfp*::*pyroA*	This study
ABPU1 *atgH*Δ	*biA1, pyrG89, wA3, argB2, pyroA4, veA1, ligD*::*ptrA, h2b-egfp*::*pyroA, atgH*::*argB*	This study
C*ypdA atgH*Δ	*biA1, pyrG89, wA3, argB2, pyroA4, veA1, ligD*::*ptrA*, P*alcA-ypdA*::*pyrG, atgH*::*argB*	This study
*sskA*Δ*srrA*Δ	*biA1, pyrG89, wA3, argB2, pyroA4, veA1, sskA*::*argB, srrA*::*argB*	Hagiwara et al., 2007a

### Construction of Gene-Replacement Cassettes

The gene-replacement cassettes for the *alcA* promoter–driven *ypdA* strain (C*ypdA*) and the *sskA* and *srrA* deletion mutants were constructed using a previously reported PCR-based fusion strategy (Yoshimi et al., 2013). The constructs for the H2B-EGFP expressing strains and the *atgH* deletion strains were constructed based on the in-fusion cloning technology as described previously (Miyazawa et al., 2018). The sequences of all primers used in this study are listed in Table 2. To construct the gene-replacement cassette for the C*ypdA* strain, a fragment containing the 5′ non-coding region of *ypdA* (amplicon 1) and one including the open reading frame of *ypdA* (amplicon 2) were PCR amplified using *A. nidulans* ABPU1 genomic DNA as a template and primers listed in Table 2 (ypdA5′-F and ypdA5′-R for amplicon 1, and ypdA-F and ypdA-R for amplicon 2). In addition, the pyrG-*alcA*(p) cassette (Yoshimi et al., 2013), which contains the *A. nidulans pyrG* marker and the *alcA* promoter (originally derived from *A. nidulans* FGSC A89), was amplified by PCR using the primers pyrG-PalcA-F and pyrG-PalcA-R (amplicon 3; Table 2). These three fragments were used as the substrates for second-round PCR fusion. The resulting major PCR product was gel-purified and used to transform the ABPU1 strain.

**Table 2:** Primer sequences used in this study.

**Purpose**	**Name**	**Sequences (5^**′**^-3^**′**^)**
Construction of the C*ypdA* strain
	ypdA5′-F	TGTAATGCCTATCAGCGTCGGC
	ypdA5′-F2	TGAATGACCTCATCGCCCCAC
	ypdA5′-R	GACAAGCACTGAGGGGGATCCACTAGTTCTAGAGCGATGTCAGTTGCTGGTCCATG
	ypdA-F	TCTTCCAATCCTATCACCTCGCCTCAAAATGGCGTCAACTACAACCAC
	ypdA-R	CTGCCACAAGGCAGTGTGCATGAG
	pyrG-PalcA-F	GCGGCCGCTCTAGAACTAGTGGATC
	pyrG-PalcA-R	GGCGAGGTGATAGGATTGGAAGAGTTCTAATTAACTG
	PalcA-357F	CGGGATAGTTCCGACCTAGGATTGGATGCA
Construction of the *sskA*Δ and *srrA*Δ strains
	sskAU-U	CAGCACCGGCGGCCGCCGGTGCTGGCTTCATTCTGC
	sskAD-D	AACCCGAAGGTACCAAGCCCAAACCTGACGATTA
	sskA-5′F	CCCATACAAGGCAAAGGCCATC
	sskA-3′R	CGCCGATACAAGACAAAACGCTG
	srrAU-U	GACTAGTTAGTGCGTTCTCCAGGATTCTTC
	srrAD-D	GGAATTCTATAACCCTCCAACTGATCCGAG
Construction of the *h2b-egfp* strains
	IF_AN7726_Fw3	CGACTCTAGAGGATCCGGGCCCCCAGAATGAAAAGCCGTCGAC
	IF_AN7726_Rv	AACTGGTACAATCGATCAATTCTTCGGTGTCGTGGG
	IF_H2B_Fw	TCGATTGTACCAGTTGGACGG
	IF_nos3′_Rv	AACCCGTATACTCCTGATCTAGTAACATAGATGACACCGCG
	IF_AnpyroA-Fw	AGGAGTATACGGGTTTTTGGC
	IF_AnpyroA-Rv	CGGTACCCGGGGATCCGGGCCCTTGGGAAGCGCAGTTGAGC
	EGFP_Rv_in	CGGGTCTTGTAGTTGCCGTC
Construction of the *atgH*Δ strains
	AoargB-F	GAATTCCATCCGCTGTGGCCGACTCA
	AoargB-R	GGTACCGACGGAGTAACTGGAAAGATACGA
	ANatgH-LU	CGACTCTAGAGGATCCGCGGCCGCCGCAGCCGCATTGGCGAAG
	ANatgH-LL+Arg	TACTCCGTCGGTACCGGCGATAGAGGCGGATATCGATAAGGAG
	ANatgH-RU+Arg	CAGCGGATGGAATTCGCCTGGCCACCGTTGTATTATCTCAC
	ANatgH-RL	CGGTACCCGGGGATCCGCGGCCGCCATCAATCGCTTTGCCGTCGTATCGAC
qRT-PCR
	Histone-RT-F	CACCCGGACACTGGTATCTC
	Histone-RT-R	GAATACTTCGTAACGGCCTTGG
	ypdA-RT-F	CGCTGGCGACTGCTAAGAAG
	ypdA-RT-R	GGATTCTTCAGGGGACGAGG
	catA-RT-F	GCTACTAGGTATCACCGCCGG
	catA-RT-R	GCTGCTCCTGGTCTGTGTGG
	hogA-RT-F	ACTACCGTGCGCCAGAAATC
	hogA-RT-R	ATCTGGAGGAGTTCCGAGAAGC

To disrupt the genes in *A. nidulans*, we replaced them with either *A. oryzae argB* or *pyroA*, as a selectable marker. To disrupt *sskA*, a plasmid containing the 1.5- and 2-kb fragments upstream and downstream of the *sskA* ORF on both sides of the *argB* gene (Hagiwara et al., 2007a) was used as a PCR template, and the primers sskAU-U and sskAD-D (Table 2) were used for PCR amplification. The resulting *sskA* disruption cassette was used to transform the C*ypdA* strain (C*ypdA*-*sskA*Δ). To construct the disruption cassette for *srrA* in the C*ypdA* strain, the same strategy was used. A plasmid containing the 1.5- and 2-kb fragments upstream and downstream of the *srrA* ORF on both sides of the *argB* gene (Hagiwara et al., 2007a) was used as a PCR template, with the primers srrAU-U and srrAD-D (Table 2). The resulting *srrA* disruption cassette was used to transform the C*ypdA* strain (C*ypdA*-*srrA*Δ). The *sskA*Δ*srrA*Δ double mutant was constructed by disruption of *sskA* in the C*ypdA*-*srrA*Δ strain, using the *sskA* disruption cassette described above (C*ypdA*-*sskA*Δ*srrA*Δ). Although *sskA* and *srrA* were disrupted by the same *argB* selectable marker, the C*ypdA*-*sskA*Δ*srrA*Δ strain is highly resistant to the phenylpyrrole-type fungicide, fludioxonil, and this fungicide could be used for selection.

To construct the *h2b-egfp*-expressing strains, a fragment containing the promoter region of *h2b* and the coding region of *h2b* with *egfp* (amplicon 1) including the terminator T*nos3*′ was PCR amplified using the plasmid pAH2BG (Maruyama et al., 2001) as a template and the primers, IF_H2B_Fw and IF_nos3′_Rv (Table 2). The gene fragment of *pyroA* (amplicon 2) and the 3′-flanking region of *pyroA* (amplicon 3) were PCR amplified using *A. nidulans* ABPU1 genomic DNA as a template and primers listed in Table 2 (IF-AnpyroA- Fw and IF-AnpyroA-Rv for amplicon 2, and IF_AN7726_Fw3 and IF_AN7726_Rv for amplicon 3). These three fragments were cloned into pUC19 using in-fusion cloning technology. The H2B-GFP expression cassette was obtained from the resulting plasmid pAH2BP by *Apa*I digestion (Supplementary Figure 7).

To disrupt *atgH, A. oryzae argB* (amplicon 1) was PCR-amplified using the plasmid for *sskA* disruption as a template and the primers AoargB-F and AoargB-R (Table 2). In addition, a fragment containing the 5′ non-coding region of *atgH* (amplicon 2) and the 3′ non-coding region of *atgH* (amplicon 3) were PCR-amplified using *A. nidulans* ABPU1 genomic DNA as a template and primers listed in Table 2 (ANatgH-LU and ANatgH-LL+Arg for amplicon 2, and ANatgH-RU+Arg and ANatgH-RL for amplicon 3). These three fragments were cloned into pUC19 using in-fusion cloning technology. The *atgH* replacement cassette was obtained by *Not*I digestion of the resulting plasmid pAAR-DatgH (Supplementary Figure 11).

### Quantitative Real-Time PCR

Real-time PCR was performed using the Mini Opticon real-time PCR system (Bio-Rad Laboratories, Hercules, CA, USA) with SYBR Green detection, according to the manufacturer's instructions. The DyNAmo SYBR Green qPCR kit (Finnzymes Oy, Espoo, Finland) was used for reaction mixture preparation. Primer sets for quantifying the expression of the *ypdA, catA*, and *hogA* genes are listed in Table 2. Primer specificity was confirmed by melting curve analysis. A cDNA sample obtained from reverse transcription of an equivalent amount of total RNA was added to each reaction mixture. The gene encoding histone H2B was used as an internal control to calculate target gene expression ratios. Relative expression ratios were calculated essentially as in Hagiwara et al. (2008). A sample from ABPU1 cells before transfer to YPD medium (0 h) was used for calibration. Relative expression ratios were calculated by first calculating the threshold cycle changes in sample and calibrator as Δ$C_{\text{t}}^{\text{sample}}$ = *C*_t(target)_ – *C*_t(histoneH2B)_ and Δ$C_{\text{t}}^{\text{calibrator}}$ = *C*_t(target)_ – *C*_t(histoneH2B)_, followed by calculating the expression ratios in both sample and calibrator as Expression ratios_sample_ = 2^Δ*Ctsample*^ and Expression ratios_calibrator_ = 2^Δ*Ctcalibrator*^, and finally, Relative expression ratios = Expression ratio_sample_ / Expression ratio_calibrator_.

### YpdA Antibody

For antibody production, we synthesized peptides corresponding to an internal region of YpdA (positions 61–74 and 159–172, AESTFDKMEKALED and TPSSPEESKKDAKA, respectively). These peptides were linked to a carrier protein and injected into a rabbit. The peptides and polyclonal antibody were prepared by Operon Biotechnology (Tokyo, Japan).

### Western Blot Analysis

Conidia (final concentration, 1 × 10^5^ conidia/ml) from the ABPU1 and C*ypdA* strains were inoculated into the indicated liquid medium and cultured for 24 h. Mycelia were frozen in liquid nitrogen, ground to powder, and immediately resuspended in protein extraction buffer containing protease inhibitors (50 mM Tris-HCl [pH 7.5], 1% sodium deoxycholate, 1% Triton X-100, 0.1% sodium dodecyl sulfate, 50 mM NaF, 5 mM sodium pyrophosphate, 0.1 mM sodium vanadate, and a protease inhibitor cocktail [Roche Applied Science, Mannheim, Germany, 1 tablet per 50 ml]). The suspension was immediately boiled for 10 min, and cell debris were removed by centrifugation for 10 min at 16,873 × g. The protein concentration of the supernatant was determined using a reducing agent–compatible Pierce BCA protein assay kit (Pierce, Rockford, IL, USA). Total protein (15 μg) was loaded onto a 12.5% polyacrylamide SDS gel, blotted onto an Immobilon-P membrane (Millipore, Bedford, MA, USA), and processed for immunodetection of YpdA using the polyclonal antibody for YpdA, HogA using the anti-Hog1 antibody (Santa Cruz Biotechnology, Inc., Santa Cruz, CA, USA), phospho-HogA using the anti-phospho-p38 MAP kinase antibody (Cell Signaling Technology, Inc., Beverly, MA, USA), and histone-H2B using the anti-histone H2B antibody (Sigma-Aldrich Japan, Tokyo, Japan), and alkaline phosphatase–coupled anti-rabbit secondary antibody (GE Healthcare, Buckinghamshire, UK).

### Microscopy

To analyze hyphal morphology and measure hyphal length between septa, conidia from the ABPU1 and C*ypdA* strains were inoculated at a final concentration of 1.5 × 10^5^ conidia/ml into liquid CDTFY and incubated statically at 37°C for 20 h (*ypdA*-inducing sample). To suppress the expression of *ypdA*, mycelia from the *ypdA*-inducing sample were transferred to a cell strainer (BD Biosciences, San Jose, CA, USA) and washed with CDY medium. The washed mycelia were transferred into CDY medium and incubated statically at 37°C for 16 h (*ypdA*-repressing sample). These *ypdA*-inducing or -repressing samples were washed with PBS (150 mM NaCl, 144 mM K_2_HPO_4_, 56 mM KH_2_PO_4_) and stained with Calcofluor white (0.1 μg/ml; MP Biomedicals, Inc., OH, USA) at room temperature for 10 min in the dark. After being washed with the same buffer, the samples were imaged under a FV1000-D IX81 confocal laser-scanning microscope (Olympus, Tokyo, Japan). Hyphal length between septa was determined using the Image J software (http://rsb.info.nih.gov/nih-image/about.html).

To analyze the number of nuclei per cell, samples of the ABPU1 and C*ypdA* strains expressing *h2b-egfp* were prepared under *ypdA*-inducing or -repressing conditions, using the method described above. For fludioxonil treatment, mycelia of the *ypdA*-inducing samples cultured in CDTFY medium for 20 h were washed with CDTFY medium and transferred into CDTFY medium containing 0.01, 0.04, or 0.1 μg/ml fludioxonil at 37°C for appropriate time (6 or 16 h). If at least five nuclei were observed in a balloon-shaped cell, it was considered to contain fragmented nuclei.

To analyze the nuclei in the freshly grown mycelia under the *ypdA*-repressing condition, germinating conidia were cultured for 8 h, then collected and transferred to CDY medium. Mycelia were sampled after 3, 6, and 9 h, and the nuclei were observed under the microscope.

### Statistical Analysis

Welch's *t*-test was used for the comparison of paired samples. The effects of two variables (strain and time) on the expression of the *ypdA, catA*, and *hogA* genes were analyzed by two-way ANOVA. Tukey–Kramer's test was used to compare multiple samples. If the treatment groups were compared with only the control group, Dunnett's test was used.

## Data Availability Statement

The original contributions presented in the study are included in the article/Supplementary Material, further inquiries can be directed to the corresponding author.

## Author Contributions

AY, DH, KF, and KA conceived and designed the experiments. YM constructed the mutant strains. YF performed transcriptional and western-blot analyses. MO performed phenotypic analysis of the mutant strains. JMaruy provided the plasmid pAH2BG and supported the nuclear distribution experiment. AY, TF, OM, NS, JMarui, YY, TN, and CT supported the experiments. AY analyzed the data. AY, KM, and KA wrote the paper. KA supervised this research and acquired funding. All authors discussed the results and commented on the manuscript.

## Conflict of Interest

The authors declare that the research was conducted in the absence of any commercial or financial relationships that could be construed as a potential conflict of interest.

## Publisher's Note

All claims expressed in this article are solely those of the authors and do not necessarily represent those of their affiliated organizations, or those of the publisher, the editors and the reviewers. Any product that may be evaluated in this article, or claim that may be made by its manufacturer, is not guaranteed or endorsed by the publisher.
