# Supplementary material for: Downregulation of the ypdA Gene Encoding an Intermediate of His-Asp Phosphorelay Signaling in Aspergillus nidulans Induces the Same Cellular Effects as the Phenylpyrrole Fungicide Fludioxonil
Source: Front Fungal Biol. 2021 Sep 1;2:675459. doi: 10.3389/ffunb.2021.675459 (PMC10512292; doi:10.3389/ffunb.2021.675459)
Supplement: Supplementary file 1 [file Data_Sheet_1.pdf]

Supplementary Materials

Downregulation of the *ypdA* gene encoding an intermediate of His-Asp phosphorelay signaling in *Aspergillus nidulans* induces the same cellular effects as the phenylpyrrole fungicide fludioxonil

Akira Yoshimi, Daisuke Hagiwara, Miyako Ono, Yasuyuki Fukuma, Yura Midorikawa, Kentaro Furukawa, Tomonori Fujioka, Osamu Mizutani, Natsuko Sato, Ken Miyazawa, Jun-ichi Maruyama, Junichiro Marui, Youhei Yamagata, Tasuku Nakajima, Chihiro Tanaka, Keietsu Abe*

*** Correspondence:** Keietsu Abe: keietsu.abe.b5@tohoku.ac.jp

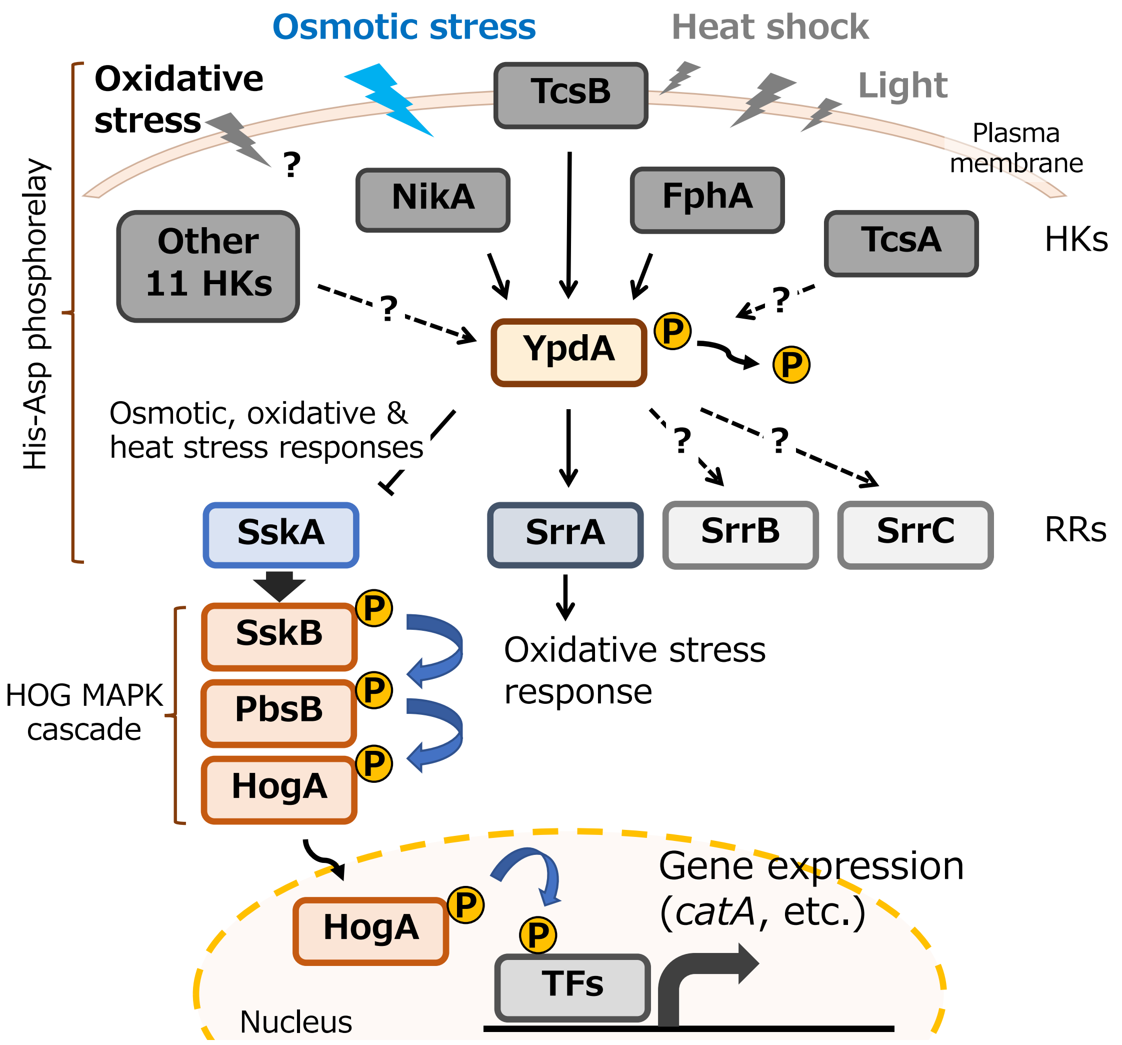

Supplementary Figure S1. Model of the two-component signaling system in *Aspergillus nidulans*. Histidine kinases (HKs) control the phosphorylation status of YpdA (His-containing phosphotransfer intermediate, HPt), and signals are transmitted to downstream response regulators (RRs). The sensor(s) of oxidative stress and the functions of other HKs are largely unknown. TFs, transcription factors.

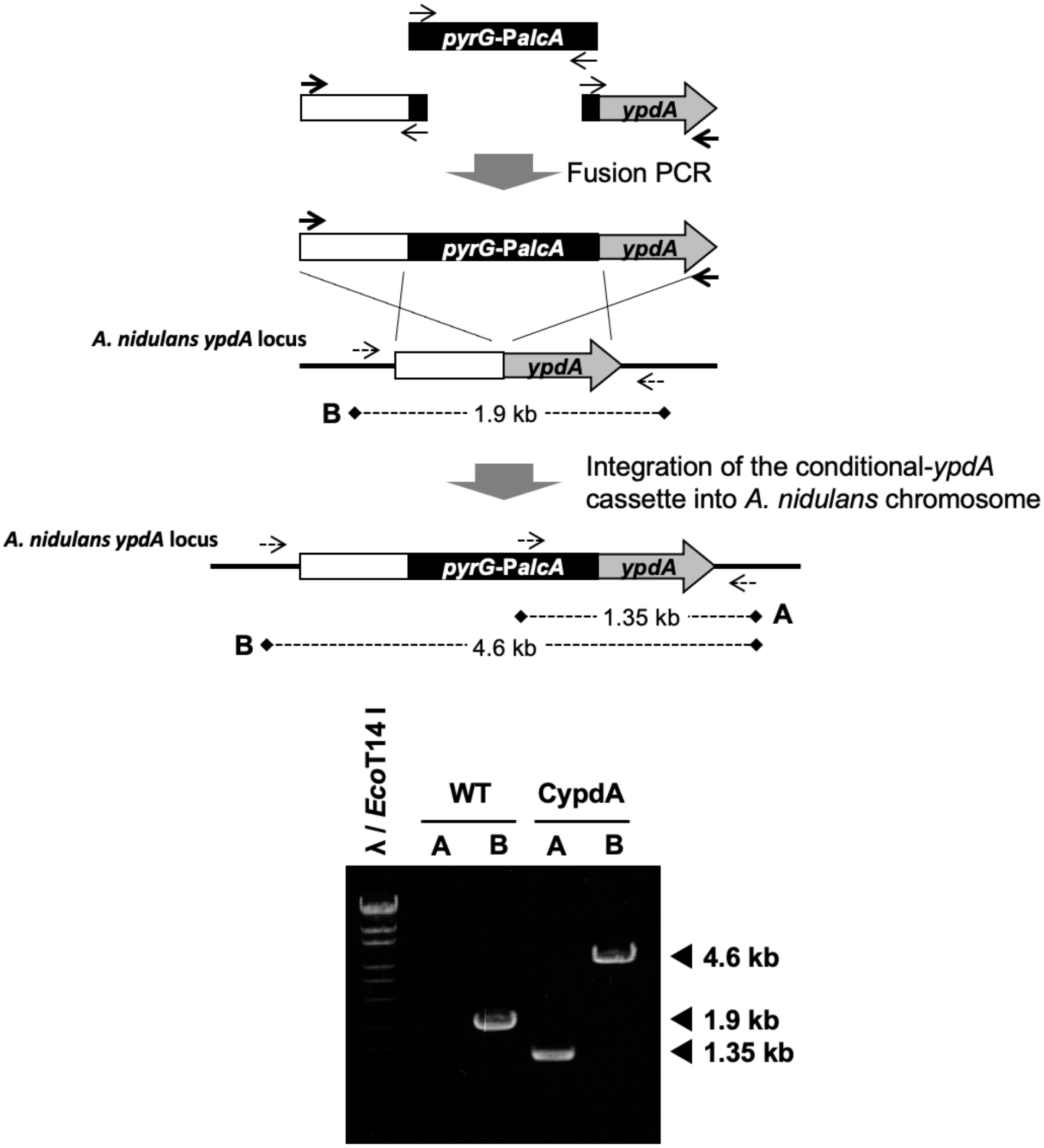

Supplementary Figure S2. Construction of the *alcA* promoter–driven *ypdA* (C*ypdA*) strain in *Aspergillus nidulans*. Schematic illustration of the *alcA* promoter–driven *ypdA* gene construction (upper panel). The first round of PCR amplified the fragments containing the right and left arms and the *pyrG*-P*alcA* cassette, which contains *pyrG* as a selectable marker and the *alcA* promoter (PalcA), for the replacement cassette. The second round of PCR fused the three separate fragments from the first round of PCR. The resulting replacement cassette was used for fungal transformation. Regions amplified by PCR for verification are indicated as A or B. PCR verification of construction of the conditional-*ypdA* strains in *A. nidulans* (lower panel). Leftmost lane, λ-*Eco*T14I digest (molecular weight marker). The primer sets (Table 2) were as follows: A, PalcA-357F and ypdA-R; B, ypdA5′-F and ypdA-R.

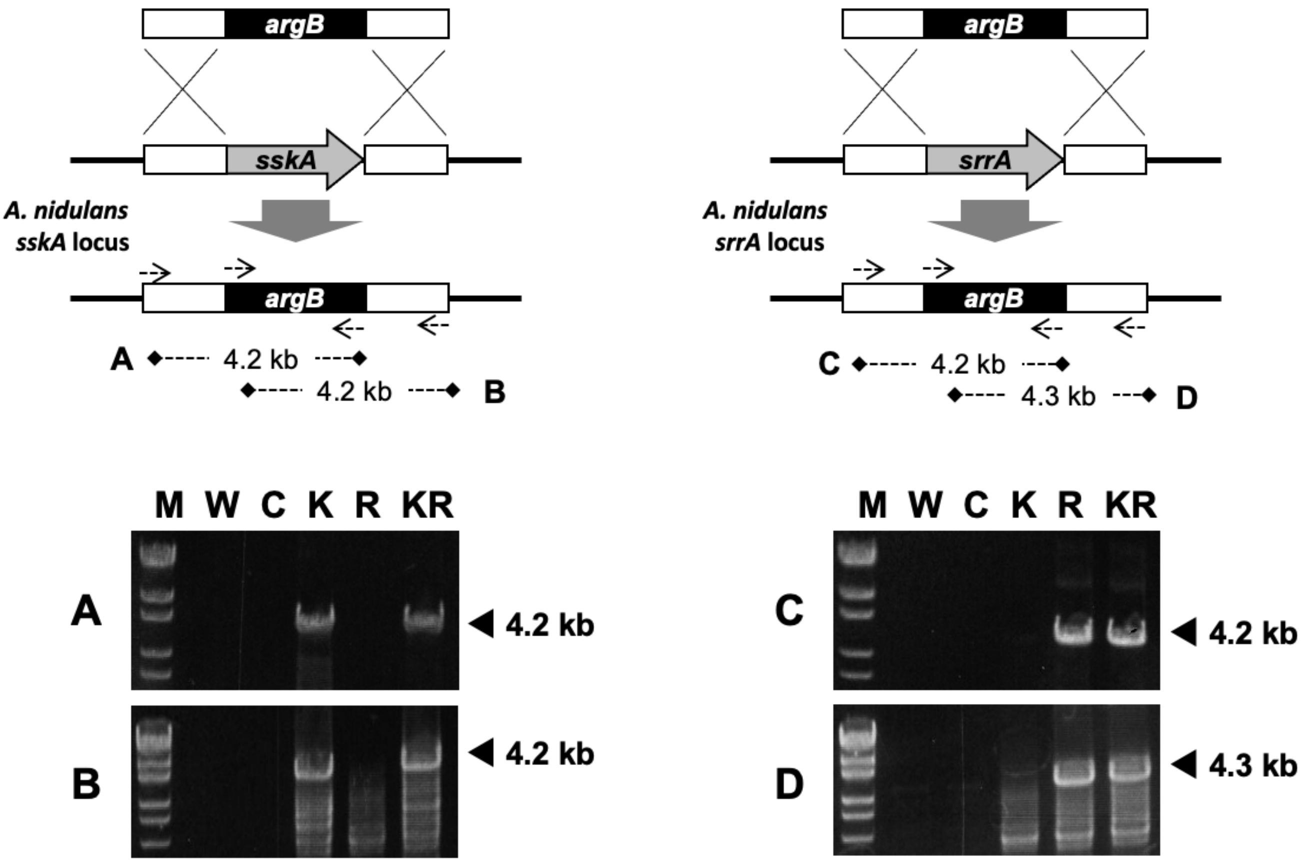

Supplementary Figure S3. Construction of *sskA* and *srrA* gene disruptant strains in *Aspergillus nidulans*. Schematic illustration of the *sskA* (left) and *srrA* (right) disruptants. Regions amplified by PCR for verification are indicated as A or B for *sskA* disruption and C or D for *srrA* disruption. M, λ-*Eco*T14I digest (molecular weight marker); W, wild-type; C, C*ypdA*; K, C*ypdA-sskA*Δ; R, C*ypdA-srrA* Δ; KR, C*ypdA-sskA*Δ*srrA*Δ. The primer sets (Table 2) were as follows: A, sskA-5′ F and AoargB-R; B, AoargB-F and sskA-3′ R; C, srrAU-U and AoargB-R; D, AoargB-F and srrAD-D.

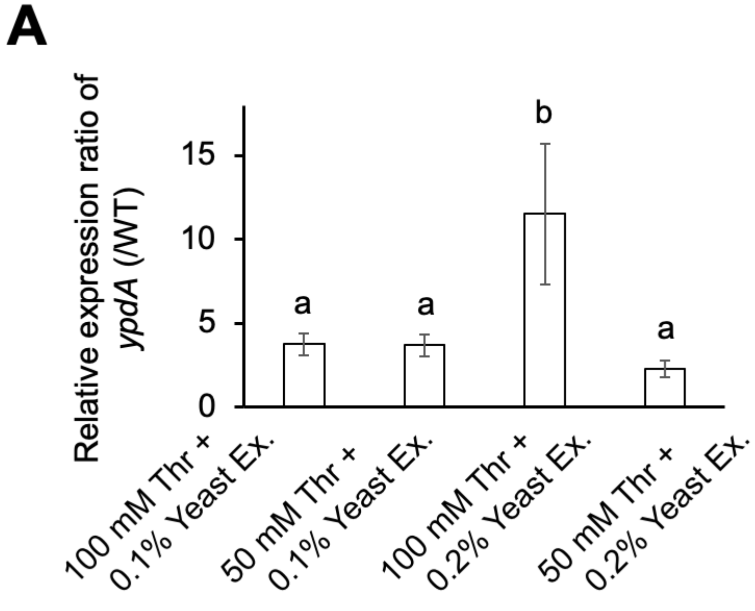

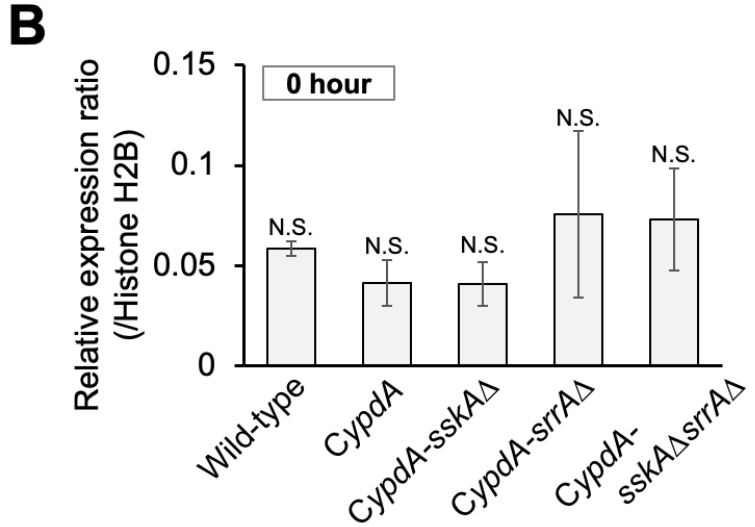

Supplementary Figure S4. Expression profile of the *ypdA* gene in C*ypdA* and its derivative strains. (A) The C*ypdA* strain was cultured in *ypdA*-inducing (CDTFY) medium (CD containing 100 or 50 mM threonine, 0.2% fructose, and 0.2% or 0.1% yeast extract instead of 0.1% glucose as a carbon source). Conidia (final concentration, 5×10^5 conidia/ml) were inoculated into the indicated liquid medium and cultured for 24 h. RNA samples of each culture were prepared, and transcription of the *ypdA* gene was quantified by RT-PCR analysis. Each value represents the ratio of transcription relative to the wild-type strain. Error bars represent the standard deviation of the mean (n = 3). Different letters indicate significant differences among media ($P < 0.05$, Tukey–Kramer's multiple comparison test). (B) Conidia (final concentration, 5×10^5 conidia/ml) of the wild-type, C*ypdA*, and C*ypdA*-derivative strains were inoculated into *ypdA*-inducing (CDTFY) medium and cultured for 20 h before transfer to *ypdA*-repressing (CDY) medium, at 0 h. RNA expression of *ypdA* was quantified by RT-PCR. Each value represents the expression ratio relative to the gene encoding histone H2B in each strain. Bars represent standard deviations. N.S., not significant.

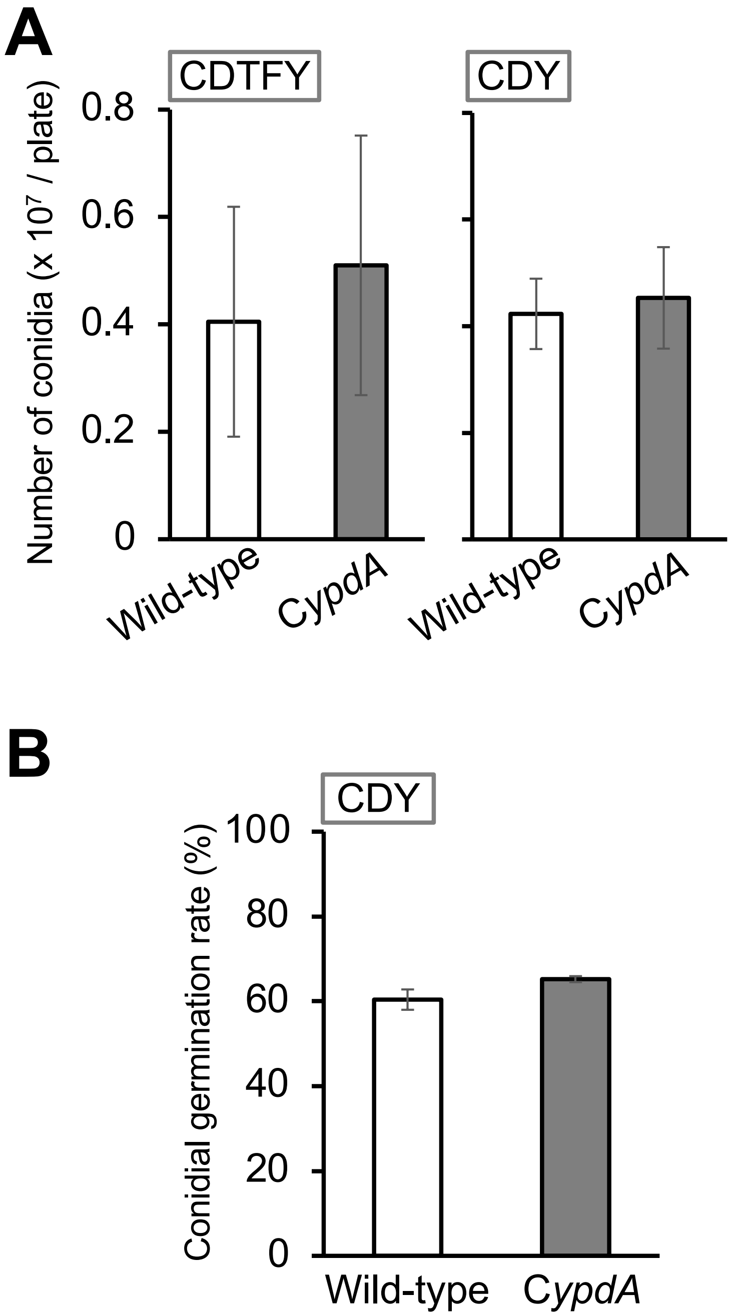

Supplementary Figure S5. Conidial formation and conidial germination rate in the wild-type and C*ypdA* strains. (A) Conidia (1 × 10^4) of each strain were spotted on 60-mm plates with *ypdA*-inducing (CDTFY) or *ypdA*-repressing (CDY) medium and cultured for 4 days at 37°C. Error bars represent standard deviations calculated from four independent replicates. (B) Conidia (final concentration, 5 × 10^6 conidia/ml) of each strain were inoculated into CDTFY medium (2 ml) and cultured for 12 h. Germinated and non-germinated conidia were counted under a microscope, and the germination rate was calculated.

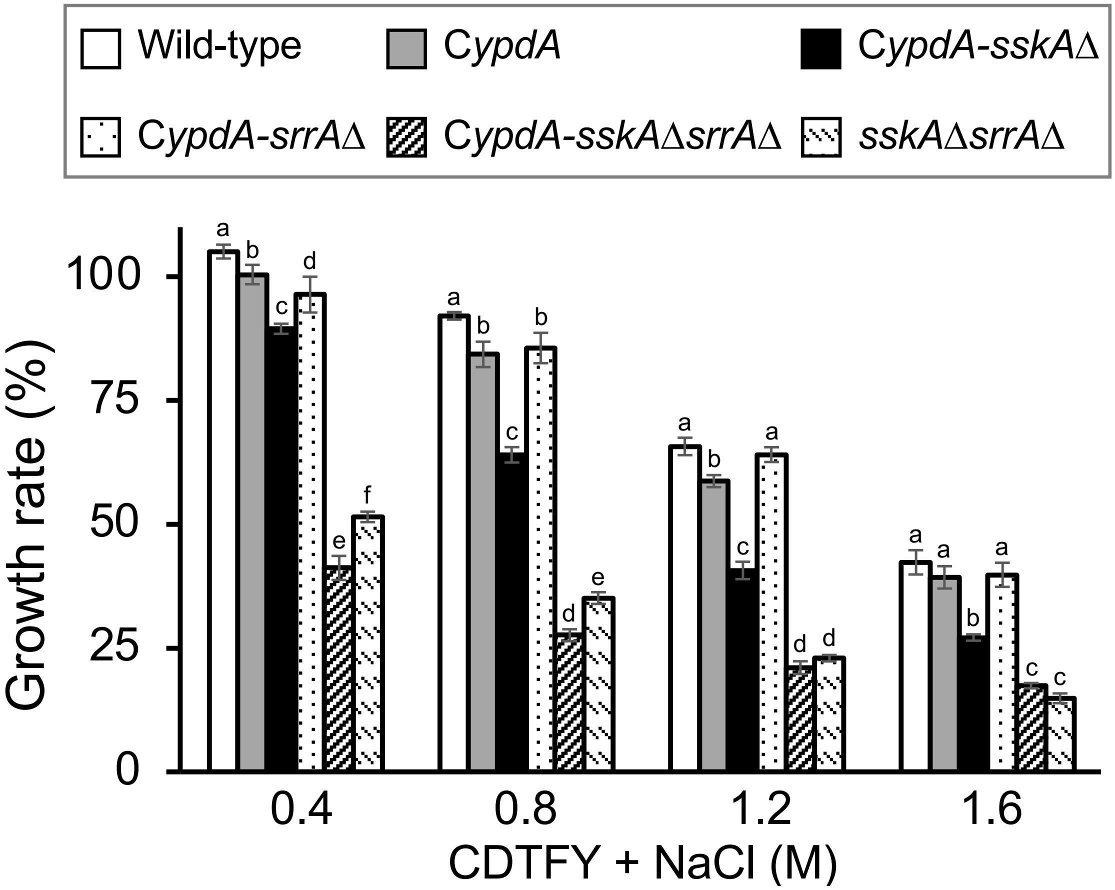

Supplementary Figure S6. Colony growth rates of the wild type, C*ypdA*, and its derivative strains on medium containing NaCl. Growth rates were determined after 4 days on *ypdA*-inducing (CDTFY) medium with the indicated concentrations of NaCl. Diameter of colonies grown without NaCl was considered 100%. Error bars represent standard deviations calculated from six independent replicates. Different letters indicate significant differences within each condition by Tukey–Kramer's test ($P < 0.05$).

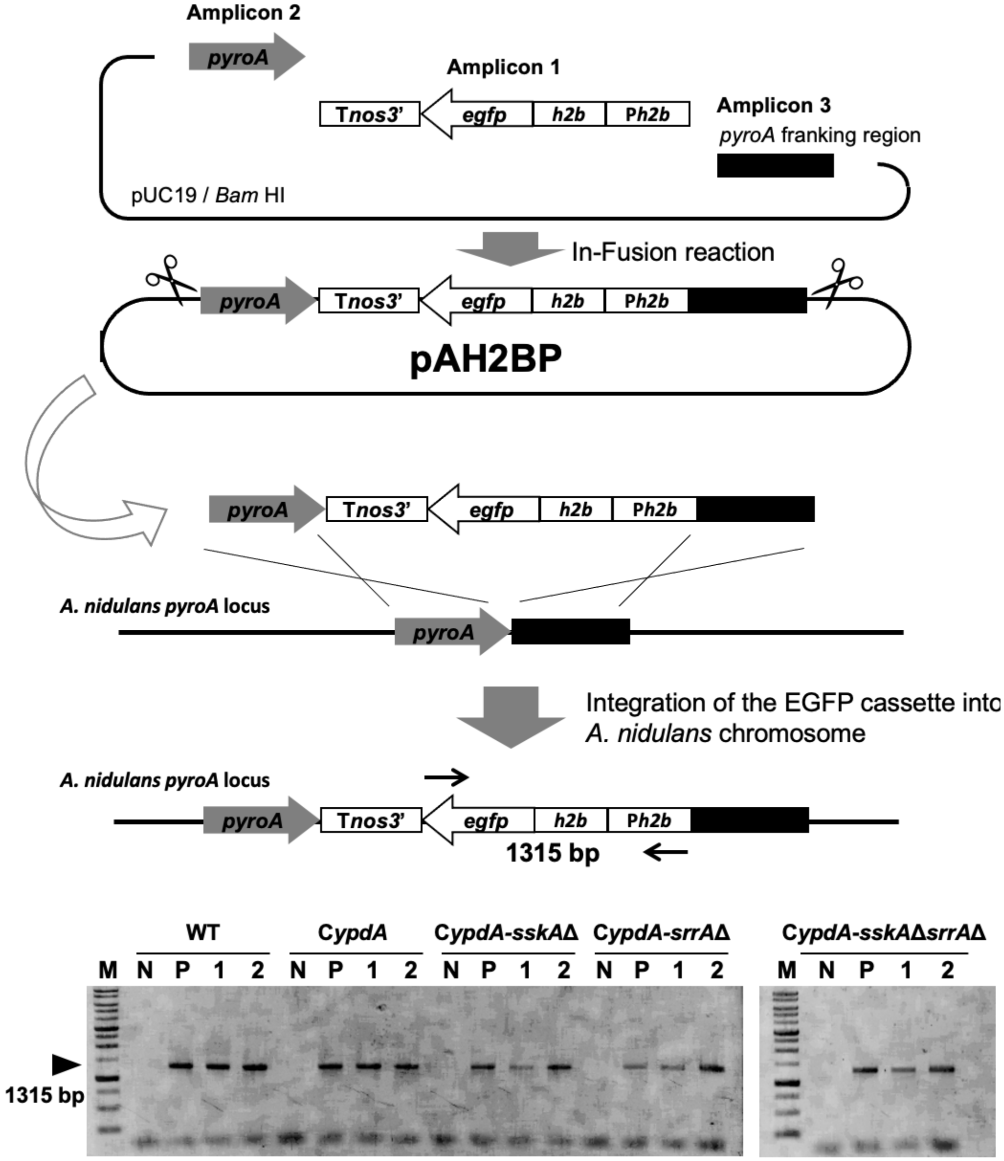

Supplementary Figure S7. Construction of *h2b-egfp*-expressing strains in C*ypdA* and its derivative strains of *Aspergillus nidulans*. Amplicon 1 (promoter region of *h2b* and coding region of *h2b with egfp* including the terminator T*nos3′*) was amplified by PCR using the plasmid pAH2BG as a template and the primers IF_H2B Fw and IF_nos3′Rv (Table 2). Amplicons 2 and 3 (*pyroA* of *A. nidulans* and the *pyroA*-flanking region, respectively) were amplified by PCR using genomic DNA of *A. nidulans* as a template and the primers IF_AnpyroA Fw and IF_AnpyroA Rv (amplicon 2) or IF_AN7726 Fw3 and IF_AN7726 Rv (amplicon 3). Fragments were purified by gel extraction and fused to *BamH*I-digested pUC19 by using an In-Fusion HD Cloning kit. The sequence of the resulting plasmid (pAH2BP) was confirmed, and a fragment containing the H2B-GFP expression cassette obtained by *Apa*I digestion of pAH2BP was used to transform the wild-type, C*ypdA*, and its derivative strains. Regions amplified by PCR for verification using the primers IF_H2B Fw and EGFP_Rv_in (Table 2) were 1315 bp (arrows). M, 100-bp ladder marker (molecular weight marker); N, negative control; P, positive control.

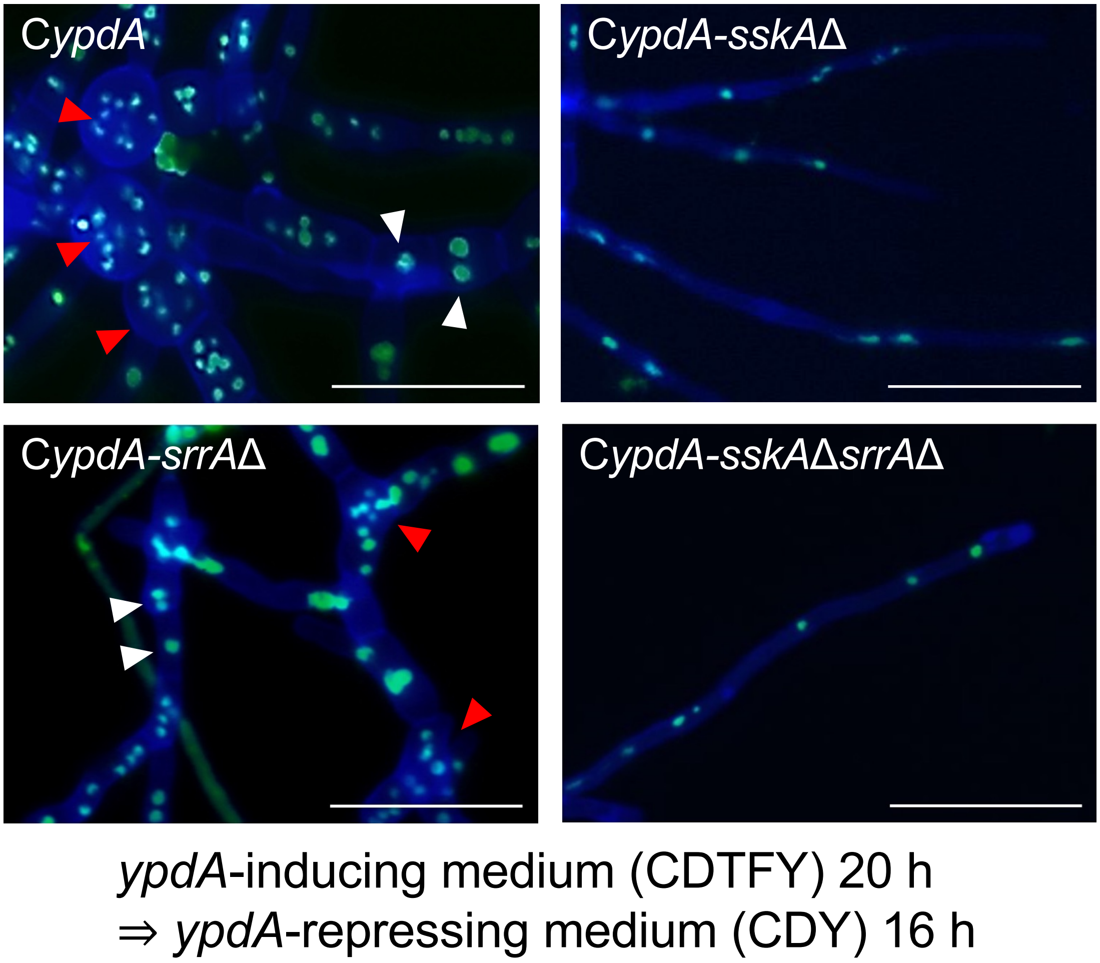

Supplementary Figure S8. Nuclear morphology of C*ypdA* and its derivative strains. Strains were cultured for 20 h in liquid *ypdA*-inducing (CDTFY) medium at 37°C and then transferred to *ypdA*-repressing (CDY) medium and incubated for 16 h at 37°C. White arrowheads indicate cells with a reduced nuclear number and red arrowheads indicate fragmented nuclei. Bars, 35 µm.

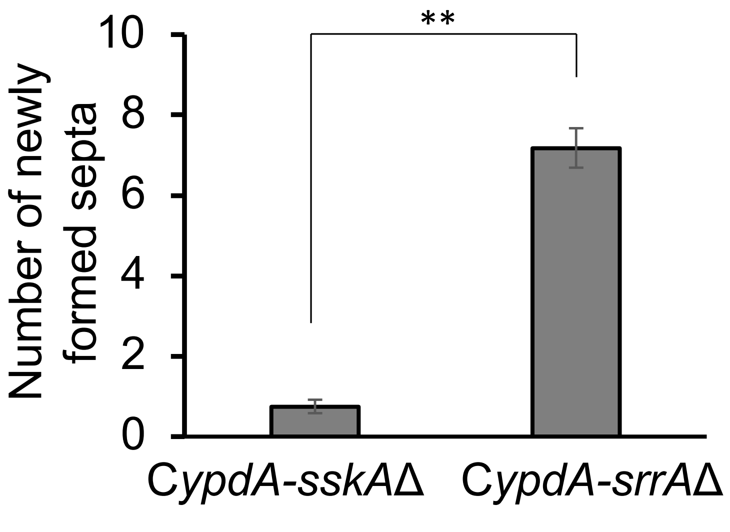

Supplementary Figure S9. Number of newly formed septa in C*ypdA* derivative strains. The number of newly formed septa in mycelia germinated from a single conidium was calculated by subtracting the average number of septa produced during preculture (CDTFY at 37°C for 10 h) from that of septa counted after incubation for 4 h after transfer to the *ypdA*-inducing medium containing 0.1 µg/ml fludioxonil (n = 100). Error bars represent standard errors. ** $P < 0.01$, Welch's t-test.

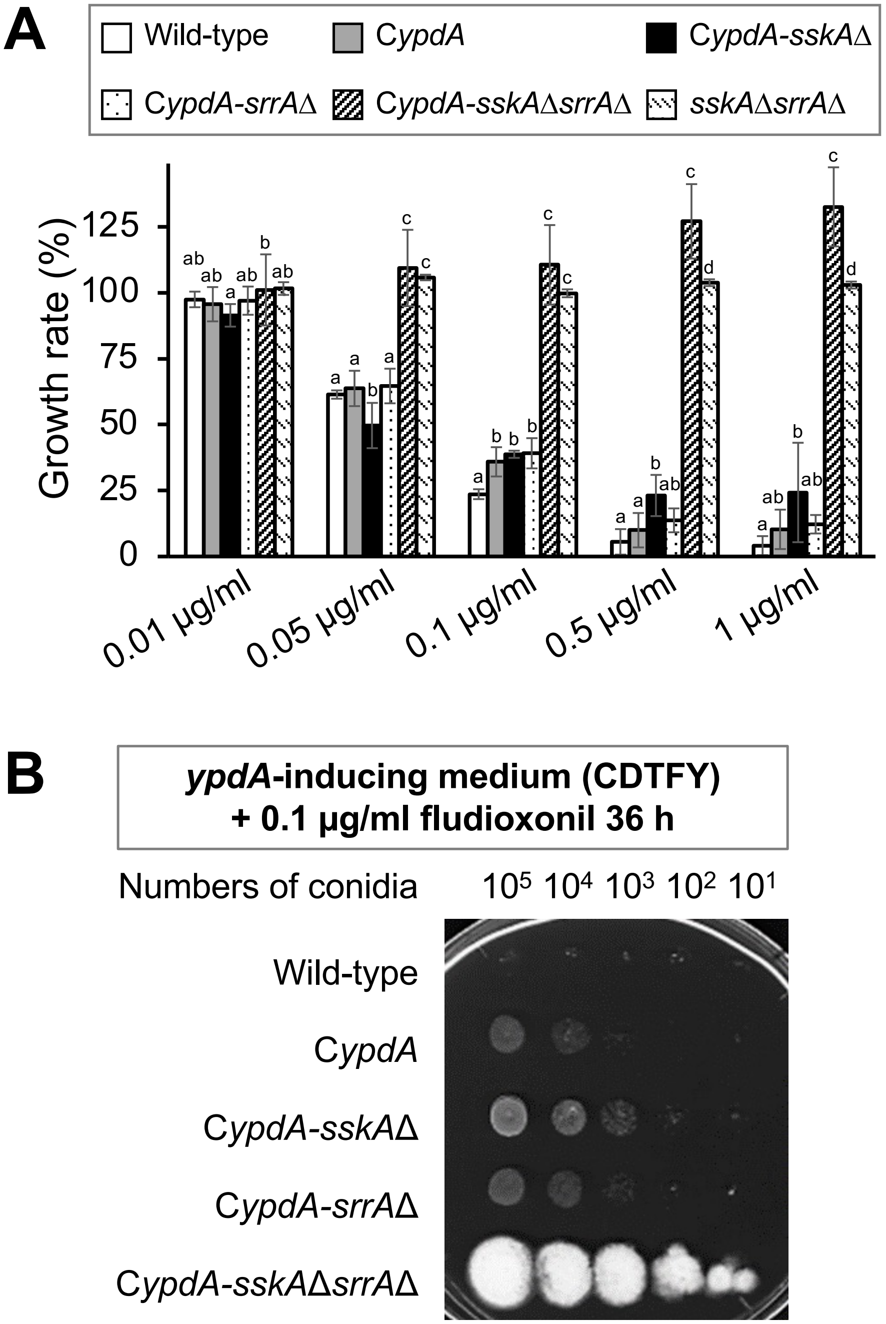

Supplementary Figure S10. Sensitivity to fludioxonil. (A) Growth rates after 4 days on *ypdA*-inducing (CDTFY) medium at the indicated concentrations of fludioxonil. Diameter of colonies grown without fludioxonil was considered 100%. The data are represented as the relative size of the colony diameter. Error bars represent standard deviations from at least six replicates. Different letters indicate significant differences within each condition by Tukey–Kramer's test ($p < 0.05$). (B) Colony growth of the wild-type, C*ypdA*, and C*ypdA*-derivative strains on fludioxonil-containing medium. The indicated numbers of conidia from the indicated strains were spotted onto CDTFY plates containing 0.1 µg/ml fludioxonil and incubated at 37°C for 36 h.

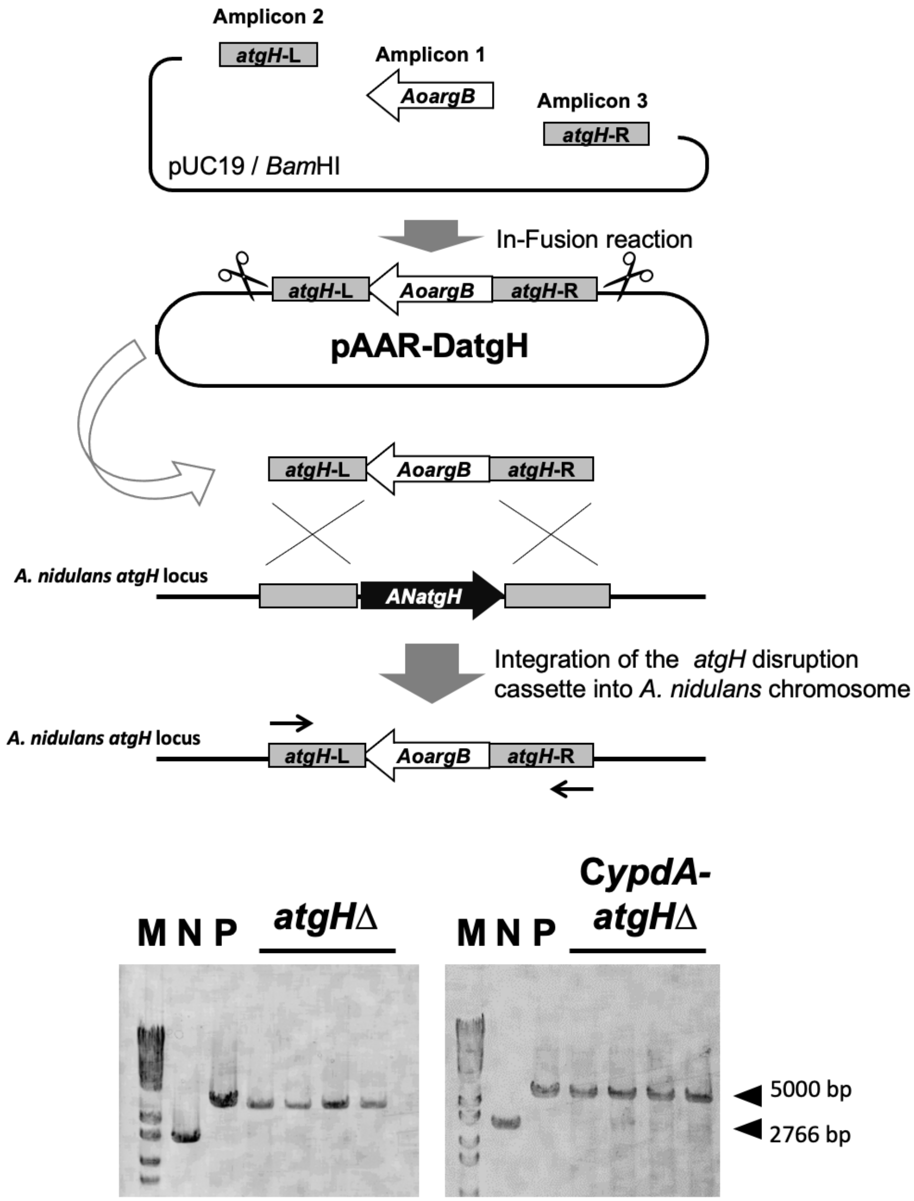

Supplementary Figure S11. Construction of *atgH* disruption strains in C*ypdA* and its derivative strains of *Aspergillus nidulans*. Amplicon 1, which contains *A. oryzae argB* as a selectable marker in *A. nidulans*, was amplified by PCR using the plasmid pAORB as a template and the primers AoargB-F and AoargB-R (Table 2). Amplicons 2 and 3, which contain the flanking region of *pyroA* of *A. nidulans*, were amplified by PCR using genomic DNA of *A. nidulans* as a template and the primers ANatgH-LU and ANatgH-LL+Arg (amplicon 2), or ANatgH-RU+Arg and ANatgH-RL (amplicon 3). Fragments were purified by gel extraction and fused to *BamH*I-digested pUC19 by using an In-Fusion HD Cloning kit. The sequence of the resulting plasmid (pAAR-DatgH) was confirmed, and the *agtH* disruption cassette obtained by *Not*I digestion of pAAR-DatgH was used to transform the wild-type and C*ypdA* strains of *A. nidulans*. PCR verification was performed using the primers ANatgH-LU and ANatgH-RL (Table 2) to amplify the regions between the arrows (5 kb if disrupted, 2.7 kb if not). M, λ-*Eco*T14I digest (molecular weight marker); N, negative control; P, positive control.

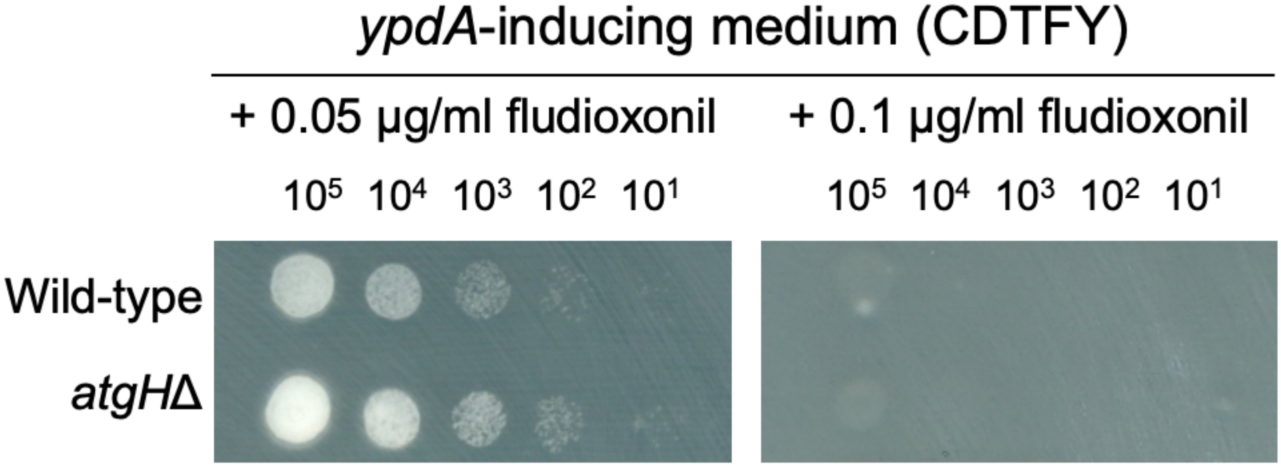

Supplementary Figure S12. Colony growth of the *Aspergillus nidulans* wild-type and *atgH*Δ strains on fludioxonil-containing medium. Serial 10-fold dilutions of conidia from the indicated strains were spotted onto CDTFY plates containing 0.05 or 0.1 μg/ml fludioxonil at the indicated final concentrations and incubated at 37°C for 36 h.

Supplementary Table S1. Relative expression ratios of the *ypdA* gene in the wild-type, C*ypdA*, and C*ypdA*-derivative strains.

Time (h)[a]	Strain	Relative expression ratio (/WT 0 h)[b]
0	Wild type	–
	C*ypdA*	0.701 ± 0.185[n.s.]
	C*ypdA-sskA*Δ	0.696 ± 0.166[n.s.]
	C*ypdA-srrA*Δ	1.332 ± 0.694[n.s.]
	C*ypdA-sskA*Δ*srrA*Δ	1.242 ± 0.443[n.s.]
12	Wild type	0.569 ± 0.102
	C*ypdA*	0.038 ± 0.020***
	C*ypdA-sskA*Δ	0.030 ± 0.016***
	C*ypdA-srrA*Δ	0.060 ± 0.011***
	C*ypdA-sskA*Δ*srrA*Δ	0.037 ± 0.010***
24	Wild type	0.579 ± 0.056
	C*ypdA*	0.101 ± 0.051***
	C*ypdA-sskA*Δ	0.038 ± 0.020***
	C*ypdA-srrA*Δ	0.118 ± 0.054***
	C*ypdA-sskA*Δ*srrA*Δ	0.048 ± 0.016***
36	Wild type	0.984 ± 0.336
	C*ypdA*	0.037 ± 0.016***
	C*ypdA-sskA*Δ	0.043 ± 0.024***
	C*ypdA-srrA*Δ	0.136 ± 0.081***
	C*ypdA-sskA*Δ*srrA*Δ	0.089 ± 0.012***

[a] Samples were prepared from mycelium at 12, 24, and 36 h after transfer from *ypdA*-inducing medium (CDTFY; 0 h) to *ypdA*-repressing medium (CDY).

[b] Because two-way ANOVA revealed a significant interaction between strain and time, multiple-comparison tests were conducted at each time point. *** $P < 0.001$ *versus* wild-type strain; Dunnett's test. n.s., not significant. Data are means ± standard deviations from three independent replicates.

Supplementary Table S2. Relative expression ratios of the *catA* gene in the wild-type, C*ypdA*, and C*ypdA*-derivative strains.

Time (h)[a]	Strain	Relative expression ratio (/WT 0 h)[b]
0	Wild type	–
	C*ypdA*	1.439 ± 0.687[n.s.]
	C*ypdA-sskA*Δ	0.596 ± 0.342[n.s.]
	C*ypdA-srrA*Δ	1.345 ± 0.686[n.s.]
	C*ypdA-sskA*Δ*srrA*Δ	1.243 ± 1.481[n.s.]
12	Wild type	1.125 ± 0.495
	C*ypdA*	53.02 ± 45.26[P = 0.0563]
	C*ypdA-sskA*Δ	0.331 ± 0.147[n.s.]
	C*ypdA-srrA*Δ	59.16 ± 29.20[*]
	C*ypdA-sskA*Δ*srrA*Δ	0.381 ± 0.286[n.s.]
24	Wild type	1.256 ± 1.039
	C*ypdA*	48.07 ± 21.00[P = 0.0509]
	C*ypdA-sskA*Δ	0.502 ± 0.402[n.s.]
	C*ypdA-srrA*Δ	68.36 ± 39.25[**]
	C*ypdA-sskA*Δ*srrA*Δ	0.591 ± 0.377[n.s.]
36	Wild type	1.740 ± 1.412
	C*ypdA*	15.68 ± 13.57[n.s.]
	C*ypdA-sskA*Δ	0.550 ± 0.343[n.s.]
	C*ypdA-srrA*Δ	58.22 ± 41.34[*]
	C*ypdA-sskA*Δ*srrA*Δ	0.931 ± 0.813[n.s.]

[a] Samples were prepared from mycelium at 12, 24, and 36 h after transfer from *ypdA*-inducing medium (CDTFY; 0 h) to *ypdA*-repressing medium (CDY).

[b] Because two-way ANOVA revealed a significant interaction between strain and time, multiple-comparison tests were conducted at each time point. [*] $P < 0.05$, [**] $P < 0.01$ or the indicated P-value *versus* wild-type strain; Dunnett's test. n.s., not significant. If the P-value is close to 0.05, the exact P-value is shown to indicate the tendency. Data are means with standard deviations from three independent replicates.

Supplementary Table S3. Relative expression ratios of the *hogA* gene in the wild-type, C*ypdA*, and C*ypdA*-derivative strains.

Time (h)[a]	Strain	Relative expression ratio (/WT 0 h)[b]
0	Wild type	–
	C*ypdA*	0.971 ± 0.230[n.s.]
	C*ypdA-sskA*Δ	0.215 ± 0.118[***]
	C*ypdA-srrA*Δ	0.950 ± 0.121[n.s.]
	C*ypdA-sskA*Δ*srrA*Δ	0.307 ± 0.144[***]
12	Wild type	0.813 ± 0.232
	C*ypdA*	1.960 ± 0.467[**]
	C*ypdA-sskA*Δ	0.179 ± 0.035[n.s.]
	C*ypdA-srrA*Δ	2.458 ± 0.588[***]
	C*ypdA-sskA*Δ*srrA*Δ	0.280 ± 0.113[n.s.]
24	Wild type	1.023 ± 0.307
	C*ypdA*	1.868 ± 0.213[**]
	C*ypdA-sskA*Δ	0.169 ± 0.058[**]
	C*ypdA-srrA*Δ	2.009 ± 0.413[**]
	C*ypdA-sskA*Δ*srrA*Δ	0.302 ± 0.195[*]
36	Wild type	0.907 ± 0.144
	C*ypdA*	1.742 ± 0.200[**]
	C*ypdA-sskA*Δ	0.170 ± 0.061[**]
	C*ypdA-srrA*Δ	1.858 ± 0.373[**]
	C*ypdA-sskA*Δ*srrA*Δ	0.310 ± 0.178[*]

[a] Samples were prepared from mycelium at 12, 24, and 36 h after transfer from *ypdA*-inducing medium (CDTFY; 0 h) to *ypdA*-repressing medium (CDY).

[b] Because two-way ANOVA revealed a significant interaction between strain and time, multiple-comparison tests were conducted at each time point. * $P < 0.05$, ** $P < 0.01$, *** $P < 0.001$ *versus* wild-type strain; Dunnett's test. n.s., not significant. Data are means ± standard deviations from three independent replicates.

Supplementary Table S4. Colony diameter of the wild-type, C*ypdA*, and C*ypdA*-derivative strains.

Strain	Medium	Colony diameter (mm) *
Wild type	CDTFY	49.06 ± 1.21[a]
C*ypdA*		48.21 ± 0.75[a]
C*ypdA-sskA*Δ		49.06 ± 0.74[a]
C*ypdA-srrA*Δ		48.46 ± 0.83[a]
C*ypdA-sskA*Δ*srrA*Δ		29.11 ± 3.34[b]
Wild type	CDY	44.17 ± 0.83[a]
C*ypdA*		30.31 ± 2.70[b]
C*ypdA-sskA*Δ		34.16 ± 1.41[c]
C*ypdA-srrA*Δ		32.08 ± 1.14[c]
C*ypdA-sskA*Δ*srrA*Δ		42.28 ± 0.94[a]

* Different letters indicate significant differences within each condition by Tukey–Kramer's test ($P < 0.01$). Data are means ± standard deviations from 14 independent replicates.

Supplementary Table S5. Distance between septa in the wild-type, C*ypdA*, and C*ypdA*-derivative strains.

Strain	Medium	Distance between septa (µm) *
Wild type	CDTFY	37.55 ± 13.06[a]
C*ypdA*		27.99 ± 8.63[b]
C*ypdA-sskA*Δ		31.78 ± 10.82[b]
C*ypdA-srrA*Δ		31.09 ± 9.10[b]
C*ypdA-sskA*Δ*srrA*Δ		42.10 ± 16.03[a]
Wild type	CDY	37.52 ± 12.60[a]
C*ypdA*		15.92 ± 8.44[b]
C*ypdA-sskA*Δ		34.65 ± 12.52[a]
C*ypdA-srrA*Δ		15.30 ± 7.08[b]
C*ypdA-sskA*Δ*srrA*Δ		47.24 ± 14.66[c]

* Different letters indicate significant differences within each condition by Tukey–Kramer's test ($P < 0.05$). Data are means ± standard deviations from the measurements of 100 cells.

Supplementary Table S6. Number of nuclei between septa in the wild-type, C*ypdA*, and C*ypdA*-derivative strains.

		Number of nuclei between septa	
**Strain**	**Medium**	**Mean ± SD***	**Median**
Wild type	CDTFY	4.32 ± 1.88[n.s.]	4
C*ypdA*		3.94 ± 1.83[n.s.]	4
C*ypdA*-*sskA*Δ		4.48 ± 2.20[n.s.]	4
C*ypdA*-*srrA*Δ		3.93 ± 1.74[n.s.]	4
C*ypdA*-*sskA*Δ*srrA*Δ		3.97 ± 2.15[n.s.]	3.5
Wild type	CDY	4.04 ± 1.92[a]	4
C*ypdA*		2.21 ± 1.55[b]	2
C*ypdA*-*sskA*Δ		4.18 ± 2.17[a]	4
C*ypdA*-*srrA*Δ		2.30 ± 1.43[b]	2
C*ypdA*-*sskA*Δ*srrA*Δ		4.78 ± 2.29[c]	5

* Different letters indicate significant differences within each condition by Tukey–Kramer's test ($P < 0.05$). Data are means ± standard deviations from the measurements of 100 cells.

Supplementary Table S7. Number of newly formed septa in the wild-type, C*ypdA*, and C*ypdA*-derivative strains.

Strain	Medium	Number of newly formed septa*
Wild type	CDTFY	3.58 ± 0.23[a]
C*ypdA*		2.81 ± 0.29[ab]
C*ypdA-sskA*Δ		2.18 ± 0.24[b]
C*ypdA-srrA*Δ		3.27 ± 0.31[a]
C*ypdA-sskA*Δ*srrA*Δ		2.17 ± 0.19[b]
Wild type	CDY	3.16 ± 0.36[a]
C*ypdA*		4.48 ± 0.46[b]
C*ypdA-sskA*Δ		1.19 ± 0.12[c]
C*ypdA-srrA*Δ		4.58 ± 0.44[b]
C*ypdA-sskA*Δ*srrA*Δ		0.96 ± 0.11[c]

* Different letters indicate significant differences within each condition by Tukey–Kramer's test ($P < 0.05$). Data are means ± standard errors from the measurements of 100 germinated conidia.

Supplementary Table S8. Cell length, number of nuclei between septa, and percentage of cells without nuclei or with fragmented nuclei in response to fludioxonil in the wild-type strain.

Fludioxonil treatment	Cell length*	Number of nuclei*	Percentage of cells	
			without nuclei	with fragmented nuclei
0.01 µg/ml for 16 h	46.20 ± 24.32[a]	4.00 ± 3.85[a]	16	0
0.04 µg/ml for 16 h	16.81 ± 5.33[b]	1.67 ± 1.03[bc]	9.1	18.2
0.1 µg/ml for 6 h	15.87 ± 4.10[b]	1.6 ± 1.67[b]	31.5	35.2
0.1 µg/ml for 16 h	14.36 ± 4.76[b]	0.29 ± 0.78[c]	59.7	28.1

* Different letters indicate significant differences within each condition by Tukey–Kramer's test ($P < 0.05$). Data are means ± standard deviations from the measurements of at least 22 cells.

Supplementary Table S9. Cell length and number of nuclei between septa, and percentage of cells without nuclei or with fragmented nuclei in the wild-type, C*ypdA*, and its derivative strains treated with 0.1 µg/ml fludioxonil for 16 h.

Strain	Cell length*	Number of nuclei*	Percentage of cells	
			without nuclei	with fragmented nuclei
Wild type	14.36 ± 4.76^{a}	0.29 ± 0.78^{a}	59.7	28.1
C*ypdA*	15.73 ± 5.21^{a}	1.79 ± 1.14^{b}	8.9	25.0
C*ypdA-sskA*Δ	19.90 ± 9.18^{a}	2.90 ± 1.87^{c}	10.9	5.5
C*ypdA-srrA*Δ	13.26 ± 5.49^{a}	0.91 ± 1.22^{ab}	40.0	24.4
C*ypdA-sskA*Δ*srrA*Δ	40.70 ± 26.52^{b}	3.54 ± 2.58^{c}	4.9	4.9

* Different letters indicate significant differences within each condition by Tukey–Kramer's test ($P < 0.05$). Data are means ± standard deviations from the measurements of at least 41 cells.

Supplementary Table S10. Number of nuclei between septa and the percentage of cells with fragmented nuclei in C*ypdA* and its derivative strains.

Strain	Number of nuclei	Percentage of cells with fragmented nuclei
C*ypdA*	2.11 ± 1.15[a]	26
C*ypdA*-*sskA*Δ	3.34 ± 1.78[b]	0
C*ypdA*-*srrA*Δ	1.88 ± 1.01[a]	14
C*ypdA*-*sskA*Δ*srrA*Δ	3.69 ± 1.92[b]	0

* Different letters indicate significant differences within each condition by Tukey–Kramer's test ($P < 0.05$). Data are means ± standard deviations from the measurements of at least 16 cells.

Supplementary Table S11. Number of vacuoles and their size in C*ypdA-atgH*Δ, *atgH*Δ, and their parental strains.

Strain	Medium	Number of vacuoles (/cell)	Vacuolar size (μm)
C*ypdA*	CDY	1.80 ± 0.76	3.33 ± 1.14
C*ypdA-atgH*Δ	CDY	1.08 ± 0.75**	2.30 ± 0.74**
Wild-type	CDTFY + 0.1 μg/ml fludioxonil	1.64 ± 0.90	5.11 ± 2.04
*atgH*Δ	CDTFY + 0.1 μg/ml fludioxonil	1.28 ± 0.67*	2.59 ± 0.68**

Data are means ± standard deviations from the measurements of 50 cells.

* $P < 0.05$, **$P < 0.01$ *versus* the corresponding parental strains; Welch's *t*-test.
